# Captive ERVWE1 triggers impairment of 5-HT neuronal plasticity in the first-episode schizophrenia by post-transcriptional activation of HTR1B in ALKBH5-m6A dependent epigenetic mechanisms

**DOI:** 10.1186/s13578-023-01167-4

**Published:** 2023-11-21

**Authors:** Xiulin Wu, Lianzhong Liu, Xing Xue, Xuhang Li, Kexin Zhao, Jiahang Zhang, Wenshi Li, Wei Yao, Shuang Ding, Chen Jia, Fan Zhu

**Affiliations:** 1 State Key Laboratory of Virology, Department of Medical Microbiology, School of Basic Medical Sciences, Wuhan University, Wuhan, 430071 China; 2 Wuhan Mental Health Center, Wuhan, 430071 China; 3 Hubei Province Key Laboratory of Allergy & Immunology, Wuhan University, Wuhan, 430071 China

**Keywords:** Schizophrenia, ERVWE1, HTR1B, ALKBH5, Synaptic plasticity

## Abstract

**Background:**

Abnormalities in the 5-HT system and synaptic plasticity are hallmark features of schizophrenia. Previous studies suggest that the human endogenous retrovirus W family envelope (ERVWE1) is an influential risk factor for schizophrenia and inversely correlates with 5-HT4 receptor in schizophrenia. To our knowledge, no data describes the effect of ERVWE1 on 5-HT neuronal plasticity. N6-methyladenosine (m6A) regulates gene expression and impacts synaptic plasticity. Our research aims to systematically investigate the effects of ERVWE1 on 5-HT neuronal plasticity through m6A modification in schizophrenia.

**Results:**

HTR1B, ALKBH5, and Arc exhibited higher levels in individuals with first-episode schizophrenia compared to the controls and showed a strong positive correlation with ERVWE1. Interestingly, HTR1B was also correlated with ALKBH5 and Arc. Further analyses confirmed that ALKBH5 may be an independent risk factor for schizophrenia. In *vitro* studies, we discovered that ERVWE1 enhanced HTR1B expression, thereby activating the ERK-ELK1-Arc pathway and reducing the complexity and spine density of 5-HT neurons. Furthermore, ERVWE1 reduced m6A levels through ALKBH5 demethylation. ERVWE1 induced HTR1B upregulation by improving its mRNA stability in ALKBH5-m6A-dependent epigenetic mechanisms. Importantly, ALKBH5 mediated the observed alterations in 5-HT neuronal plasticity induced by ERVWE1.

**Conclusions:**

Overall, HTR1B, Arc, and ALKBH5 levels were increased in schizophrenia and positively associated with ERVWE1. Moreover, ALKBH5 was a novel risk gene for schizophrenia. ERVWE1 impaired 5-HT neuronal plasticity in ALKBH5-m6A dependent mechanism by the HTR1B-ERK-ELK1-Arc pathway, which may be an important contributor to aberrant synaptic plasticity in schizophrenia.

**Supplementary Information:**

The online version contains supplementary material available at 10.1186/s13578-023-01167-4.

## Introduction

Schizophrenia, characterized by positive, negative, and cognitive symptoms [1], is a severe psychiatric illness with a lifetime morbidity of approximately 1% [2]. Generally, schizophrenia manifests in late adolescence or early adulthood, leading to unemployment rates of 80–90% [3], which causes high levels of family burden. Understanding the underlying mechanisms of schizophrenia is crucial for effective treatment. Recent genome-wide association studies (GWAS) have successfully identified common genetic variations in genes encoding synaptic proteins, glutamate receptors, dopamine receptors, and 5-hydroxytryptamine (5-HT, also known as serotonin) receptors that are associated with schizophrenia [4, 5]. Furthermore, postmortem studies have provided evidence that disturbances in 5-HT receptors are involved in the pathogenesis of schizophrenia [6].

5-HT1B receptor (HTR1B), one subfamily of 5-HT receptors, regulates 5-HT release from the 5-HT terminals and memory processing. Higher transcript levels of HTR1B have been reported in schizophrenia [6, 7]. As a G-protein-coupled receptor (GPCR), HTR1B activates the extracellular signal-regulated kinase (ERK) signal pathway in the postsynaptic membrane [8–10]. Activated ERK plays an essential role in regulating the synthesis of Activity regulated cytoskeleton associated protein (Arc) [11–13], a protein primarily enriched in the postsynaptic membrane that is critical for maintaining synaptic plasticity [14, 15]. Dysregulation of synaptic plasticity, characterized by structural changes, including decreased dendritic arbor complexity and reduced dendritic spine density [16], participates in the pathophysiology of schizophrenia. In addition to HTR1B, other 5-HT receptors, including HTR1A [7], HTR2A [7], HTR3 [17], HTR4 [18, 19], and HTR6 [20] are associated with schizophrenia. Intriguingly, our recent study demonstrates that ERVWE1, a significant risk factor in schizophrenia, can inhibit HTR4 expression and contribute to the etiology of schizophrenia [18].

Human endogenous retroviruses (HERVs) are relics of infectious retroviruses in our ancestors, constituting approximately 8% of the human genome [21]. A HERV provirus structure consists of gag, pro, pol, env, and two long terminal repeats (LTRs), which harbor regulatory functions including promoter, enhancer, or primer-binding sites [22]. HERVs have long been regarded as “genomic dark matter”. However, accumulating evidence now reveals their involvement in various biological processes, such as antiviral immunity, cellular differentiation, cellular gene expression, and genomic recombination through viral proteins, promoter and enhancer elements derived from the long retroviral terminal repeats, or HERV-derived long non-coding RNA (lncRNA) [23, 24]. Many factors, including viral infection [25–27], drugs [28], and epigenetic modifications [29], can activate HERV elements. HERV-W, one HERV family, is also known as multiple sclerosis (MS) associated retrovirus (MSRV). The envelope protein of HERV-W, referred to as ERVWE1, Syncytin-1, or HERV-W env, plays a role in embryonic trophoblast formation, immunomodulatory activity during pregnancy, and embryonic innate antiviral immunity [30]. Significant expression of ERVWE1 has been observed in various diseases, including cancers [31, 32], autoimmune diseases [33], and neuropsychiatric disorders [34, 35]. Notably, data from our lab has confirmed that ERVWE1 contributes to the occurrence and progression of schizophrenia by increasing the expression of schizophrenia-associated genes [35], activating ion channels [18], modulating mitochondrial respiratory chain function [36], impairing synaptic function and spine development [37, 38], and inducing neuroinflammatory abnormalities [39–42].

Epigenetic modifications, which participate in energy metabolism [43], neuronal differentiation [44], synaptic transmission [45], and immune responses [46], play a meaningful role in the etiology and pathophysiology of schizophrenia [47]. Among epigenetic modifications, RNA modification has emerged as an important regulatory layer of gene expression. One reversible RNA methylation modification, known as m6A modification, is the most common post-transcriptional mRNA modification in mammals, adding a new dimension to our understanding of post-transcriptional regulation of gene expression. Quite a few findings indicate that m6A can regulate gene expression by influencing mRNA metabolism, stability, and translation [48]. The installation of m6A modification is mediated by “writers”, METTL3/14/16, WTAP, KIAA1429 (VIRMA), RBM15/15B, and ZC3H13, recognized by “readers” proteins, such as YTHDF1/2/3, YTHDC1/2, and IGF2BP1/2/3, and obliterated by “eraser” including ALKBH5 and FTO [49]. Abnormalities in m6A modification are associated with cancers [50] and neurological diseases [51]. However, no literature reports have explored whether ERVWE1 undergoes m6A modification, which could potentially influence synaptic plasticity.

Herein, higher levels of HTR1B, Arc, and ALKBH5 were detected in schizophrenia patients compared with the healthy controls and had a positive correlation with ERVWE1. Interestingly, HTR1B also showed positive correlations with ALKBH5 and Arc in schizophrenia. Further analysis revealed that ALKBH5 was a possible independent risk factor for schizophrenia. Additionally, in *vitro* studies revealed that ERVWE1 upregulated HTR1B expression and impaired 5-HT neuronal plasticity through the ERK-ELK1-Arc signaling pathway. Moreover, our results demonstrated that ERVWE1 led to a decrease in the global m6A level. The upregulation of HTR1B induced by ERVWE1 depended on ALKBH5-mediated demethylation. Importantly, ALKBH5 was involved in the impairment of 5-HT neuronal plasticity caused by ERVWE1. Mechanistically, ERVWE1 upregulated HTR1B through ALKBH5-mediated m6A-dependent epigenetic modifications, contributing to the impairment of 5-HT neuronal plasticity by activating the ERK-ELK1-Arc signal pathway in schizophrenia. Our findings revealed a novel risk gene in schizophrenia and a new mechanism by which ERVWE1 regulated 5-HT neuronal plasticity through epigenetic modifications, providing insights into the role of ERVWE1 in the etiology of schizophrenia through the RNA modification pathway.

## Results

### Bioinformatics analysis demonstrated abnormality of the HTR1B signal pathway in the brain of schizophrenia

**Fig. 1:**
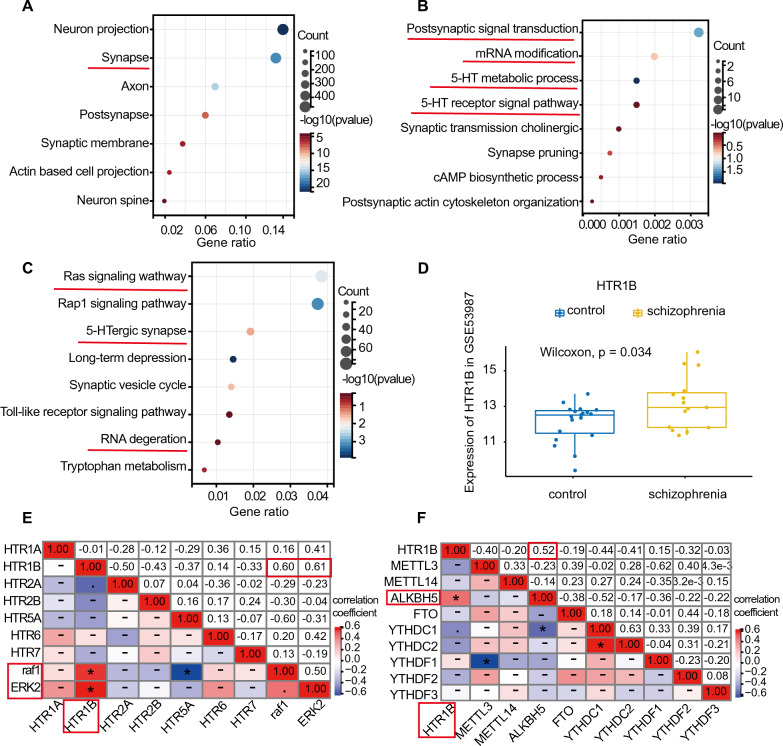
Higher expression of HTR1B in schizophrenia was associated with ERK2 and m6A modification in GSE53987. **A**, **B** Partial visualization of GO (Gene Ontology) analysis for DEGs, **A** GOCC analysis, **B** GOBP analysis, X-axis represents gene ratio, Y-axis represents different ontologies, the circle color represents *p*-value and the circle size shows count number. **C** KEGG (Kyoto Encyclopedia of Genes and Genomes) pathway enrichment analysis for DEGs. **D** Boxplot of HTR1B expression in the prefrontal cortex (BA46) of schizophrenia patients (*n* = 15) and healthy controls (*n* = 19), *p*-value by Wilcoxon (Mann-Withney). **E** Correlation heat map analysis among 5-HT receptors, raf1 and ERK2, red and blue represent positive and negative correlation, *p < 0.05 by Spearman. **F** Correlation matrix analysis of HTR1B and m6A modification-related genes, red represents positive and blue represents negative correlation, *p < 0.05 by Spearman. The correlation coefficients are shown in the corresponding grids. Data are mean ± SD

Schizophrenia is a common psychiatric disorder with a complex etiology. Extensive research has shown that dysregulation of several neurotransmitters, including dopamine [38], glutamate [52], and 5-HT [53] may contribute to the development of schizophrenia. Microarray analysis is commonly used to identify differentially expressed genes (DEGs) and signal pathways. In this study, we utilized the GSE53987 dataset to analyze DEGs in the postmortem prefrontal cortex (BA46) of individuals with schizophrenia and healthy controls. GOCC (Gene Ontology Cell Component) analysis revealed that the DEGs were mainly associated with synapses (Fig. 1A). Furthermore, GOBP (Gene Ontology Biological Process) analysis indicated that these DEGs were involved in postsynaptic signal transduction, mRNA modification, and 5-HT receptor signal pathway (Fig. 1B). KEGG (Kyoto Encyclopedia of Genes and Genomes) analysis further demonstrated that the DEGs were enriched in the Ras signaling pathway, 5-HTergic synapse, and RNA degradation (Fig. 1C). There are seven types of 5-HT receptors (HTR1-7) [54]. Importantly, the analysis of DEGs revealed an elevated level of HTR1B in the prefrontal cortex (PFC) (BA46) of individuals with schizophrenia compared to healthy controls (*p* = 0.034) (Fig. 1D), while the expression of HTR2A was significantly decreased (*p* = 0.005) (Additional file 1: Fig. S1A). However, we did not observe a significant pattern of gene expression alteration in other 5-HT receptors, including HTR1A (*p* = 0.15), HTR2B (*p* = 0.089), HTR5A (*p* = 0.3), HTR6 (*p* = 0.083), and HTR7 (*p* = 0.24) (Additional file 1: Fig. S1B–F). These findings support the presence of aberrant expression of 5-HT receptors in schizophrenia.

Based on the abnormalities observed in the Ras pathway in our KEGG analysis, we investigated whether alterations in 5-HT receptors were associated with raf1 and ERK2, two critical members of the Ras pathway. The correlation heatmap results revealed a significant positive association between HTR1B and raf1, as well as ERK2 (Fig. 1E, p < 0.05, correlation coefficient = 0.6, and 0.61, respectively). As our GOBP analysis indicated abnormal mRNA modification in schizophrenia, we also examined the correlation between HTR1B and m6A modification-related genes, which are the most prevalent mRNA modification. Intriguingly, we discovered a positive correlation between HTR1B and the m6A demethylase ALKBH5 in schizophrenia patients (*p* < 0.05, correlation coefficient = 0.52, Fig. 1F), suggesting that aberrant expression of HTR1B might be associated with m6A demethylation. In conclusion, we proposed that HTR1B was subjected to m6A modification and associated with the ERK pathway in schizophrenia based on our bioinformatics analysis.

### Aberrant expression of HTR1B, Arc, and ALKBH5 displayed a strong positive correlation with ERVWE1 in the blood of first-episode schizophrenia

**Fig. 2:**
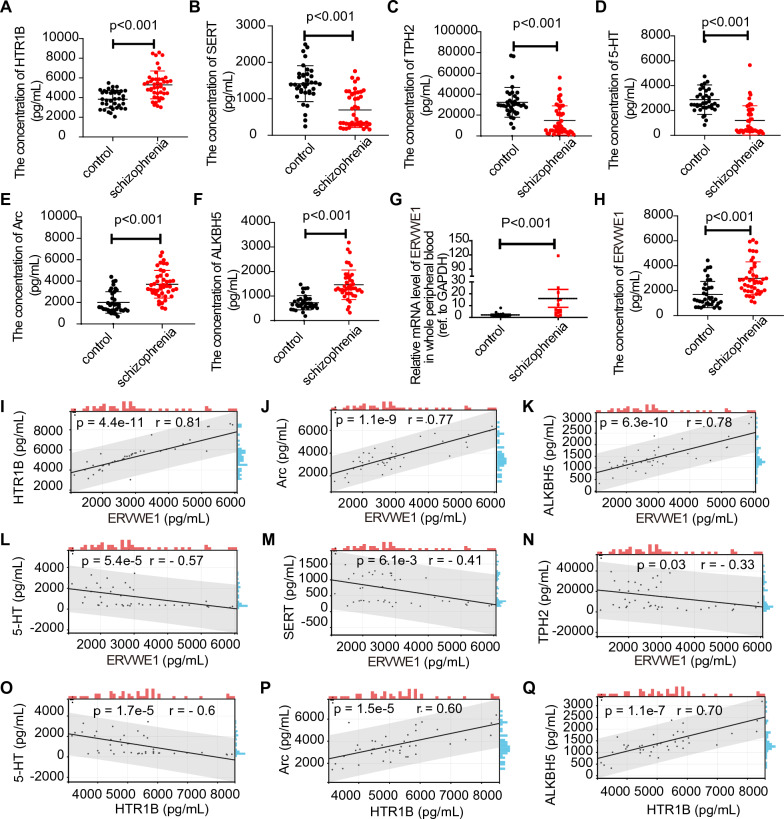
Abnormal 5-HTergic systems and HTR1B signal pathway in the blood of schizophrenia. **A**-**F** The concentrations of HTR1B, SERT, TPH2, 5-HT, Arc, and ALKBH5 in the plasma of schizophrenia patients (*n* = 44) and controls (*n* = 37) by ELISA (*p* < 0.001 by median and nonparametric analysis). **G** The mRNA level of ERVWE1 in the whole peripheral blood of schizophrenia patients (*n* = 15) and healthy controls (*n* = 14) by RT-qPCR (*p* < 0.001 by median and nonparametric analysis). **H** Differences in ERVWE1 protein expression in schizophrenia patients (*n* = 44) and controls (*n* = 37) by ELISA (*p* < 0.001 by median and nonparametric analysis). **I**–**N** Correlation of ERVWE1 protein level with HTR1B (r = 0.81, *p* < 0.001, *n* = 44), Arc (r = 0.77, *p* < 0.001, *n* = 44), ALKBH5 (r = 0.78, *p* < 0.001, *n* = 44), 5-HT (r = – 0.57, *p* < 0.001, *n* = 44), SERT (r = – 0.41, *p* < 0.01, *n* = 44), and TPH2 (r = − 0.33, *p* = 0.03, *n* = 44) protein levels in the plasma of schizophrenia patients by Spearman. **O**–**Q** Correlation analysis of plasma HTR1B with plasma 5-HT (r =  − 0.6, *p* < 0.001, *n* = 44), Arc (r = 0.6, *p* < 0.001, *n* = 44), and ALKBH5 (r = 0.7, *p* < 0.001, *n* = 44) in schizophrenia by Spearman. Dots depict schizophrenia patients. Data are mean ± SD, but a few are overlapping and cannot be separated on the graph

Given the bioinformatics findings indicating abnormalities in 5-HT receptors in the brains with schizophrenia, we investigated the levels of 5-HT receptors in the blood of first-episode schizophrenia. We observed a significant increase in both the mRNA expression level (*p* < 0.05, Additional file 1: Fig. S2A) and plasma protein concentration of HTR1B (*p* < 0.001, Fig. 2A and Table 1) in first-episode schizophrenia. However, there were no significant differences in the mRNA expression levels of HTR1A (*p* = 0.17), HTR2A (*p* = 0.09), HTR6 (*p* = 1.0), and HTR7 (*p* = 0.476) (Additional file 1: Fig. S2B–E) between schizophrenia patients and healthy controls. Furthermore, both the mRNA and protein levels of the 5-HT transporter (SERT) (*p* < 0.001 in mRNA level and *p* < 0.001 in protein level, Additional file 1: Fig. S2F, Fig. 2B, and Additional file 1: Table S1) and the 5-HT synthesis enzymes tryptophan hydroxylase-2 (TPH2) (*p* < 0.05 in mRNA and *p* < 0.001 in protein levels, Additional file 1: Fig. S2G, Fig. 2C, and Additional file 1: Table S2) were significantly lower in first-episode schizophrenia patients compared to the healthy controls. Additionally, we found a decrease in 5-HT levels in schizophrenia (*p* < 0.001, Fig. 2D and Additional file 1: Table S3), indicating dysregulation of the 5-HT system in schizophrenia. HTR1B is known to play a critical role in synaptic plasticity [55]. To assess whether the increase in HTR1B expression is associated with synaptic plasticity, we measured the level of Arc, a plasticity-related gene. We observed a significant increase in both the mRNA (*p* < 0.01, Additional file 1: Fig. S2H) and protein (*p* < 0.001, Fig. 2E and Table 2) levels of Arc in schizophrenia. Moreover, our bioinformatics analysis revealed a positive correlation between HTR1B and ALKBH5. Consistently, we found elevated levels of ALKBH5 in both the whole peripheral blood mRNA (*p* < 0.01, Additional file 1: Fig. S2I) and plasma protein (*p* < 0.001, Fig. 2F and Table 3) in schizophrenia, indicating m6A modification dysfunction in schizophrenia.

**Table 1:** The concentration of HTR1B in the plasma of healthy controls and schizophrenia patients

Healthy controls (*n* = 37, pg/mL)		schizophrenia patients (*n* = 44, pg/mL)	
Mean	3841.2973	Mean	5291.7273
Median	3844	Median	5234
Std. Deviation	891.05633	Std. Deviation	1413.24114
Skewness	− 0.123	Skewness	0.731
Sta. Error of Skewness	0.388	Sta. Error of Skewness	0.357
Range	3420	Range	5560
Minimum	2064	Minimum	3024
Maximum	5484	Maximum	8584

**Table 2:** The concentration of Arc in the plasma of healthy controls and schizophrenia patients

Healthy controls (*n* = 37, pg/mL)		schizophrenia patients (*n* = 44, pg/mL)	
Mean	2007.2278	Mean	3703.5844
Median	1590.8571	Median	3522.2857
Std. Deviation	1005.01614	Std. Deviation	1288.2449
Skewness	0.942	Skewness	0.48
Sta. Error of Skewness	0.388	Sta. Error of Skewness	0.357
Range	3702.86	Range	5268.57
Minimum	699.43	Minimum	1419.43
Maximum	4402.29	Maximum	6688

**Table 3:** The concentration of ALKBH5 in the plasma of healthy controls and schizophrenia patients

Healthy controls (*n* = 37, pg/mL)		schizophrenia patients (*n* = 44, pg/mL)	
Mean	728.8378	Mean	1460.7727
Median	651	Median	1316
Std. Deviation	289.36122	Std. Deviation	601.56381
Skewness	0.941	Skewness	0.934
Sta. Error of Skewness	0.388	Sta. Error of Skewness	0.357
Range	1290	Range	2860
Minimum	186	Minimum	321
Maximum	1476	Maximum	3181

In line with our previous studies [18, 35–40], we found higher levels of ERVWE1 in schizophrenia patients (Fig. 2G, H and Additional file 1: Table S4). Subsequent analysis revealed several positive correlations between ERVWE1 and HTR1B (*p* = 0.01, r = 0.65, Additional file 1: Fig. S2J), Arc (*p* = 0.03, r = 0.57, Additional file 1: Fig. S2K), and ALKBH5 (*p* < 0.01, r = 0.78, Additional file 1: Fig. S2L) in the mRNA levels of schizophrenia patients, as determined by Spearman's correlation. Furthermore, the median analysis demonstrated that the plasma protein level of HTR1B (+) (HTR1B > 4138.3903 pg/mL) was detected in 29 out of 31 ERVWE1 (+) patients (ERVWE1 > 2043.8078 pg/mL), while the plasma protein level of HTR1B (−) (HTR1B ≤ 4138.3903 pg/mL) was detected in 8 out of 13 ERVWE1 (−) patients (ERVWE1 ≤ 2043.8078), suggesting a consistent expression pattern of HTR1B and ERVWE1 in schizophrenia patients (Table 4). Consistent with the correlation analysis in mRNA levels, we found ERVWE1 had a positive correlation with HTR1B (*p* < 0.001, r = 0.81, Fig. 2I), Arc (*p* < 0.001, r = 0.77, Fig. 2J), and ALKBH5 (*p* < 0.001, r = 0.78, Fig. 2K) in the protein levels of schizophrenia patients, in which correlation coefficient all reached 0.7 or above, implying that there was a very robust correlation among them. Furthermore, we showed that higher level of ERVWE1 inversely correlated with 5-HT level (*p* < 0.001, r = − 0.57, Fig. 2L) in schizophrenia patients. However, ERVWE1 had weak negative correlations with SERT and TPH2 in schizophrenia patients (Fig. 2M, N). Strikingly, plasma HTR1B concentration also correlated significantly with plasma 5-HT, Arc, and ALKBH5 concentration in schizophrenia patients (r = − 0.6 in 5-HT, r = 0.6 in Arc, r = 0.7 in ALKBH5, Fig. 2O–Q), indicating that HTR1B was associated with m6A modification in schizophrenia. Additionally, the univariate and multivariate analyses showed that ALKBH5 was an independent risk factor for schizophrenia (Table 5). Taking together, higher levels of HTR1B and ALKBH5 in the blood of schizophrenia patients positively correlated with ERVWE1, and ALKBH5 was a novel schizophrenia risk gene.

**Table 4:** The consistency of ERVWE1 and HTR1B concentration in schizophrenia patients

Schizophrenia patients	ERVWE1 (+)	ERVWE1 (-)	Consistency ratio
HTR1B (+)	29	5	84%
HTR1B (–)	2	8	

ERVWE1 (+): the expression of ERVWE1 above 2043.8078 pg/mL; ERVWE1 (–): the expression of ERVWE1 below 2043.8078 pg/mL; HTR1B (+): the expression of HTR1B above 4138.3903 pg/mL; HTR1B (–): the expression of HTR1B below 4138.3903 pg/mL

**Table 5:** Univariate and multivariate analysis of risk factors for schizophrenia

Characteristics	Univariate (*p*)	Multivariate			
		OR	95% CI		*p*
			Lower	Upper	
Gender	0.873				NA
Age	0.619				NA
Smoking	0.996				NA
BMI	0.480				NA
Education	0.608				NA
ERVWE1 protein level	0.001	3.725	1.156	11.995	0.028
ALKBH5 protein level	< 0.001	20.244	5.589	73.325	< 0.001
HTR1B protein level	0.002				NS
Arc protein level	< 0.001				NS

NS, *p* > 0.05

*NA* not adopted

### ERVWE1 could upregulate HTR1B and Arc expression

**Fig. 3:**
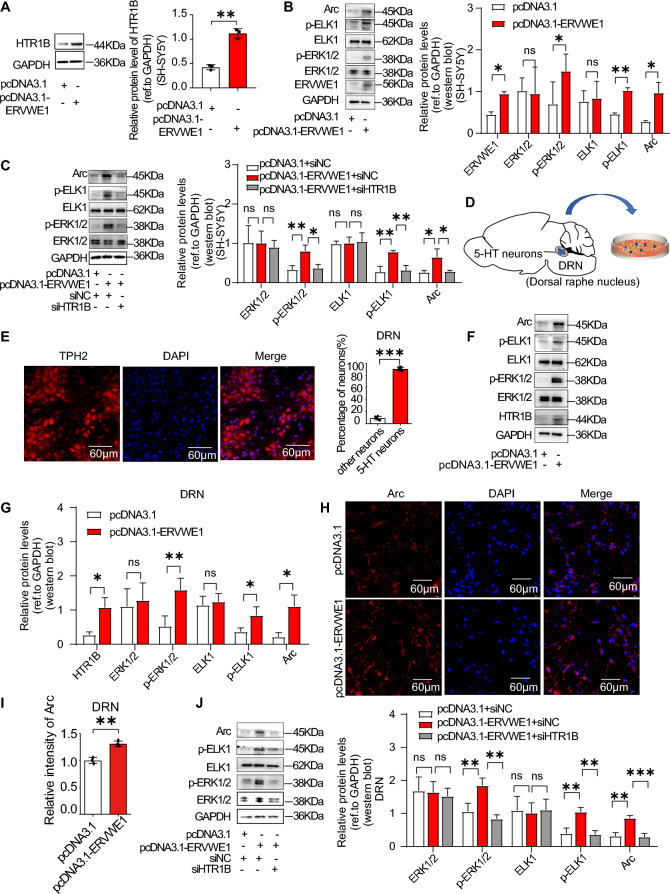
ERVWE1 upregulated HTR1B leading to ERK-ELK1-Arc signaling pathway activation. **A** Representative western blot result of HTR1B in SH-SY5Y cells. **B** ERVWE1 increased p-ERK1/2, p-ELK1, and Arc protein levels in SH-SY5Y cells. **C** The relative protein levels of ERK1/2, p-ERK1/2, ELK1, p-ELK1, and Arc when co-transfected with ERVWE1 (or empty plasmid) and siHTR1B (or siNC) in SH-SY5Y cells. **D** The diagrammatic drawing for 5-HT neurons isolated from the DRN of newborn Sprague–Dawley Rats (P0–P7). **E** TPH2 immunostaining showed the percentage of 5-HT neurons in DRN neurons (*n* = 5 rats per group). **F**, **G** Western blot results of HTR1B and downstream proteins expression levels after transfection with ERVWE1 in DRN neurons. **H**, **I** Immunofluorescence staining with Arc in DRN neurons. **J** DRN neurons were transfected with ERVWE1 and siHTR1B. Data are mean ± SD. Statistical analysis: Student’s *t*-test and one-way ANOVA (^ns^ *p* > 0.05, *p < 0.05, **p < 0.01, ***p < 0.001). All experiments were repeated 3 times

To further investigate the relationship between ERVWE1 and HTR1B in schizophrenia, we transfected ERVWE1 into the human neuroblastoma cell line SH-SY5Y (Additional file 1: Fig. S3A, B), which is commonly used to study neuropsychiatric disorders [56], to gain a deeper understanding of their association. ERVWE1 significantly increased the protein level of HTR1B in SH-SY5Y cells (Fig. 3A). HTR1B is known to activate the ERK pathway [9, 10], so we aimed to establish the role of HTR1B in the ERK-ELK1-Arc pathway. As expected, HTR1B amplification enhanced the levels of p-ERK1/2 (Thr202/Tyr204) and p-ELK1 (S383) and Arc protein, while not affecting ERK1/2 and ELK1 levels (Additional file 1: Fig. S4A, B), indicating that HTR1B activation regulated p-ERK1/2 (Thr202/Tyr204), p-ELK1 (S383) and Arc expression. Conversely, HTR1B knockdown using HTR1B specific siRNA suppressed the phosphorylation of ERK1/2 and ELK1, as well as reduced Arc expression (Additional file 1: Fig. S4C, D), suggesting that HTR1B could activate the ERK-ELK1-Arc pathway. Considering that ERVWE1 upregulated HTR1B, we investigated the effects of ERVWE1 on the ERK-ELK1-Arc pathway. Our results demonstrated that ERVWE1 promoted the activation of the ERK-ELK1-Arc pathway (Fig. 3B). Remarkably, depletion of HTR1B by RNA interference abolished the upregulation of p-ERK1/2, p-ELK1, and Arc induced by ERVWE1 in SH-SY5Y cells (Fig. 3C and Additional file 1: Fig. S5), confirming that HTR1B was a key mediator of the ERK-ELK1-Arc pathway in response to ERVWE1.

HTR1B is one of the 5-HT receptors and can be expressed in 5-HTergic neurons and non-5-HTergic neurons. To study the effect of ERVWE1 on 5-HTergic neurons, we isolated the dorsal raphe nucleus (DRN), which has a high proportion of 5-HTergic neurons, from Sprague–Dawley rats (P0-P7) for further study (Fig. 3D). To confirm the presence of cultured neurons containing 5-HT neurons, we labeled TPH2, a marker specific to 5-HT neurons, and found that almost 90% of neurons were TPH2^+^ neurons (Fig. 3E). We also genetically expressed ERVWE1 in the DRN neurons (Additional file 1: Fig. S6A) and validated that ERVWE1 led to an increase in HTR1B, p-ERK1/2, p-ELK1, and Arc in 5-HT neurons using western blot (Fig. 3F, G). Similarly, the immunofluorescence density of HTR1B and Arc qualitatively increased (Additional file 1: Fig. S6B and Fig. 3H, I), suggesting that ERVWE1 could enhance HTR1B and Arc levels in 5-HT neurons. Importantly, the increased expression of p-ERK, p-ELK1, and Arc induced by ERVWE1 was abolished in HTR1B-deficient neurons (Fig. 3J and Additional file 1: Fig. S6C), indicating the involvement of HTR1B in the ERVWE1-regulated pathway. Overall, ERVWE1 elevated HTR1B expression and upregulated Arc by activating the ERK-ELK1-Arc signal pathway.

### ERVWE1 decreased dendritic complexity and spine density in 5-HT neurons

**Fig. 4:**
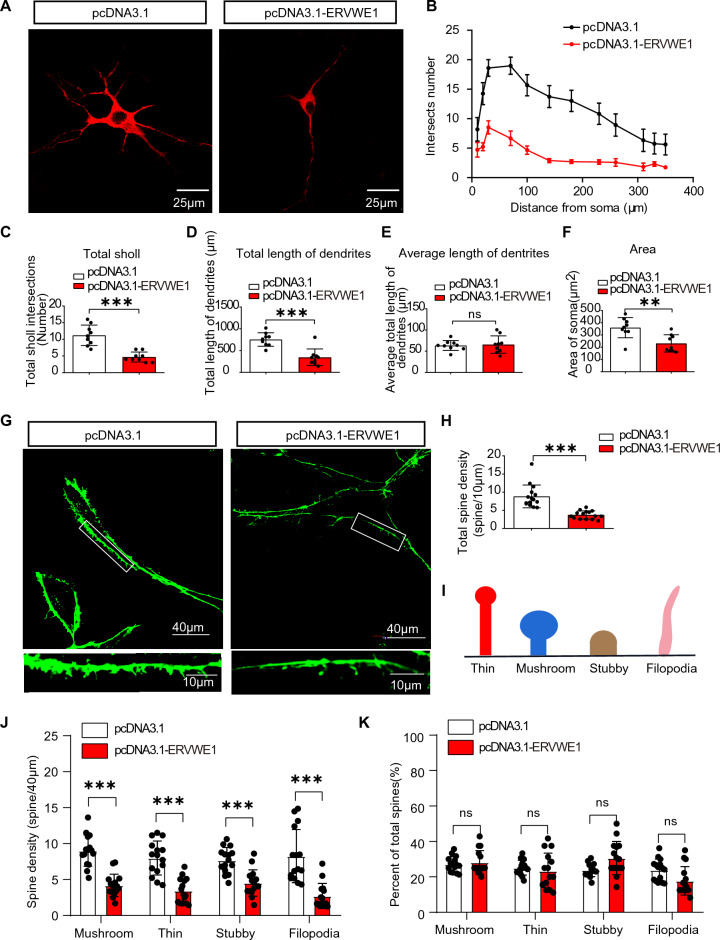
ERVWE1 decreased 5-HT neurons complexity and spine density. **A** Morphology of 5-HT neurons stained with MAP2 (red) in control and ERVWE1 groups. Scale bars = 25 μm. **B**–**F** The Sholl analysis of 5-HT neurons in control and ERVWE1 groups. **B** The intersection numbers (*n* = 9), **C** Total dendritic branching numbers (*n* = 9), **D** Total dendritic length (*n* = 9), **E** Average dendritic length (*n* = 9), and **F** soma area (*n* = 9). **G**–**K** ERVWE1 decreased spines density of 5-HT neurons. **G** Confocal images of 5-HT neurons transfected with control and ERVWE1 expressed vectors, Scale bars = 40 μm / 10 μm, **H** Total spine density (*n* = 15), **I** A schematic diagram to show four types of spines, **J** Spine density of each subtype (*n* = 15), and **K** Percentage of total spine density in four subtypes (*n* = 15). Data are mean ± SD. Statistical analysis: Student’s *t*-test (^ns^ *p* > 0.05, **p < 0.01, ***p < 0.001)

Arc, which is enriched in neuronal dendrites, has been extensively reported to be associated with weak synaptic plasticity [14, 57, 58]. We genetically overexpressed ERVWE1 in DRN neurons for several days, achieving a transfection efficiency of approximately 40% (Additional file 1: Fig. S7). Our results demonstrated that ERVWE1 decreased the complexity of 5-HT neurons, as indicated by immunostaining with MAP2 (a typical neuronal marker) using a confocal microscope (Fig. 4A). Compared to the control groups, ERVWE1 significantly reduced the numbers of dendritic branches (Fig. 4B, C) and the total length of dendrites (Fig. 4D) in 5-HT neurons, while the average length of dendrites showed no significant changes (Fig. 4E). Additionally, we observed a decrease in the soma size of 5-HT neurons (Fig. 4F). These findings suggested that the high level of ERVWE1 was associated with a reduction in dendritic arborization, leading to decreased complexity of 5-HT neurons.

Alterations in spine density have been linked to synaptic plasticity, and it has been shown that reduced spine density is associated with schizophrenia, possibly due to excessive spine pruning during late childhood or adolescence [59]. Therefore, we investigated the effect of ERVWE1 on dendritic spine architecture. ERVWE1 significantly reduced the total spine density of 5-HT neurons compared to the controls (Fig. 4G, H). Based on their morphology, dendritic spines can be categorized into four subtypes: mushroom, stubby, thin, and filopodia (Fig. 4I). In the presence of ERVWE1, there was a significant decrease in density of all four spine types (Fig. 4J). However, the proportion of spine subtypes did not change (Fig. 4K). Our findings indicated that the increased levels of ERVWE1 in 5-HT neurons were associated with lower spine density. In conclusion, ERVWE1 played a critical role in dendritic morphogenesis, suggesting that ERVWE1 might contribute to schizophrenia by affecting synaptic plasticity.

### HTR1B involvement in ERVWE1 regulated neuronal complexity and spine density

**Fig. 5:**
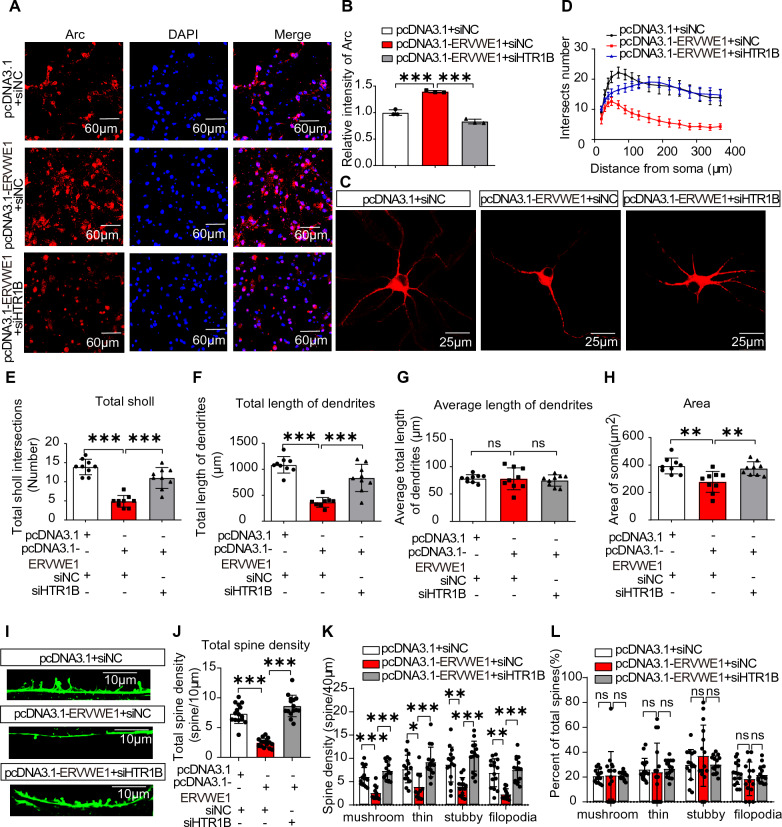
HTR1B participated in ERVWE1-regulated 5-HT neuronal plasticity.** A**, **B** The relative fluorescence intensity of Arc. Scale bars = 60 μm. **C** The morphology of 5-HT neurons. Scale bars = 25 μm. **D**–**H** The dendrites branching number, total number, total length, average length, and soma area of 5-HT neurons after co-transfection with ERVWE1 and siHTR1B (*n* = 9). **I**–**L** The spine density in the pcDNA3.1 + siNC, pcDNA3.1-ERVWE1 + siNC, and pcDNA3.1-ERVWE1 + siHTR1B groups. **I**, **J** Total spine density (*n* = 15), Scale bars = 10 μm, **K** Each subtype’s spine density (*n* = 15), **L** Percentage density of each spine subtypes (*n* = 15). Data are mean ± SD. Statistical analysis: one-way ANOVA (^ns^ *p* > 0.05, **p < 0.01, ***p < 0.001)

Our cytological experiment revealed that ERVWE1 upregulated the expression of HTR1B and Arc. Remarkably, the deficiency of HTR1B inhibited the elevated immunoreactivity of Arc induced by ERVWE1 in 5-HT neurons (Fig. 5A, B). Considering the impact of ERVWE1 on 5-HT plasticity, we investigated the role of HTR1B in regulating 5-HT plasticity. Surprisingly, the loss of HTR1B expression resulted in a more pronounced increase in the numbers of dendritic branches (Fig. 5C–E), and the total length of dendrites (Fig. 5F), while the average dendritic length showed no significant changes (Fig. 5G) compared to the group exposed to ERVWE1 alone. Moreover, applying short-interfering HTR1B rescued the reduction of 5-HT neuron soma size caused by ERVWE1 (Fig. 5H). These findings suggested that ERVWE1 impaired 5-HT neuronal plasticity through HTR1B mediation.

Immunolabeling of FITC Phalloidin in 5-HT neurons revealed that ERVWE1 decreased the total spine density, and this effect was absent when HTR1B was silenced (Fig. 5I, J). Further analysis of individual dendritic spine classes confirmed that HTR1B deficiency prevented the decrease in spine density mediated by ERVWE1 not only in mature spines (mushroom and stubby spines) but also in immature spines (thin and filopodia spines) (Fig. 5K). However, there were limited changes in the proportion of spines (Fig. 5L), indicating the involvement of HTR1B in the reduction of spine density induced by ERVWE1. The consistent results suggested that ERVWE1 weakened 5-HT plasticity by activating the HTR1B signal pathway to regulate Arc expression in schizophrenia.

### ERVWE1 induced HTR1B upregulation in an ALKBH5-m6A-dependent manner

**Fig. 6:**
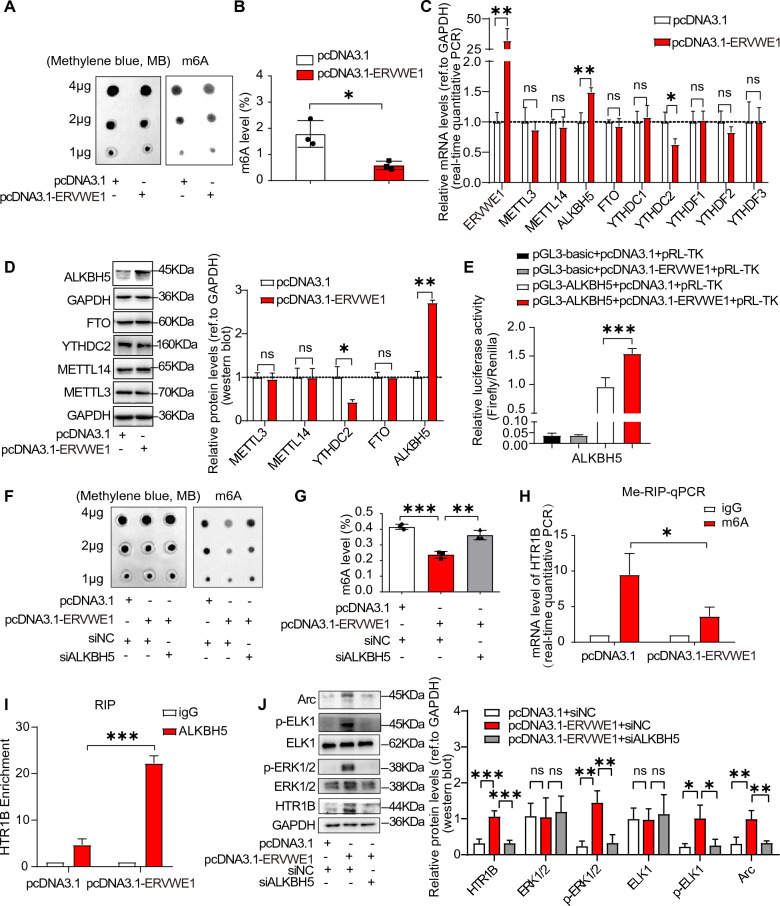
ERVWE1 reduced the global m6A level dependent ALKBH5 contributed to HTR1B upregulation. **A**, **B** The global m6A level was measured by **A** dot blot, and **B** the EpiQuik m6A RNA Methylation Quantification Kit (Colorimetric) in ERVWE1 expressed groups in SH-SY5Y cells. Methylene blue (MB) was leveraged as a control. **C** The relative mRNA levels of methyltransferase (METTL3 and METTL14), demethylases (ALKBH5 and FTO), and reader proteins (YTHDC1, YTHDC2, YTHDF1, YTHDF2, and YTHDF3) in the control and ERVWE1 groups by RT-qPCR in SH-SY5Y cells. **D** The western blot results of METTL3, METTL14, ALKBH5, FTO, and YTHDC2 in SH-SY5Y cells. **E** ALKBH5 promoter activity as measured by Promega Dual-Luciferase Reporter Assay. **F**, **G** The global m6A levels were measured by dot blot and the EpiQuik m6A RNA Methylation Quantification Kit after co-transfection with ERVWE1 (or empty plasmid) and siALKBH5 (or siNC) in SH-SY5Y cells. **H** m6A level of HTR1B was determined by Me-RIP-qPCR assay after overexpression of ERVWE1 in SH-SY5Y cells. **I** ERVWE1 increased the enrichment of ALKBH5 in HTR1B mRNA by RIP-qPCR analysis in SH-SY5Y cells. **J** The expression levels of HTR1B and downstream proteins were detected by western blot when ERVWE1 was co-transfected with siALKBH5 or siNC in SH-SY5Y cells. Data are mean ± SD. Statistical analysis: Student’s *t*-test (two groups) and one-way ANOVA (three groups) (^ns^ *p* > 0.05, *p < 0.05, **p < 0.01, ***p < 0.001). All experiments were repeated 3 times

Clinical results revealed a positive correlation between ERVWE1 and ALKBH5, an important demethylase of m6A modification. Therefore, we investigated the effect of ERVWE1 on m6A modification. We observed a significant decrease in the overall level of m6A methylation in SH-SY5Y cells treated with ERVWE1, as demonstrated by dot blot and RNA methylation quantification analysis (Fig. 6A, B). RT-qPCR and western blot showed a substantial increase in ALKBH5 levels upon exposure to ERVWE1 (Fig. 6C, D). Although ERVWE1 decreased YTHDC2 expression, ALKBH5 showed the highest abundance. Additionally, we conducted a Dual-Luciferase Reporter Assay to explore how ERVWE1 regulated ALKBH5 expression. We found that ERVWE1 could enhance ALKBH5 promoter activity (Fig. 6E), suggesting that ERVWE1 increased ALKBH5 expression by modulating its promoter activity. Interestingly, we found that ALKBH5 dynamically regulated global m6A levels when it was overexpressed or disrupted (Additional file 1: Fig. S8A, B). Additionally, the dot blot and RNA methylation quantification analysis demonstrated that ALKBH5 deficiency rescued the decreased m6A level induced by ERVWE1 (Fig. 6F, G), indicating that ERVWE1 reduced m6A levels through ALKBH5-mediated demethylation.

Subsequently, we performed methylated RNA immunoprecipitation qPCR (Me-RIP-qPCR) to investigate whether HTR1B mRNA underwent m6A modification. Our results showed that HTR1B was a direct target of m6A modification (Fig. 6H). Importantly, ERVWE1 reduced the HTR1B mRNA m6A level, suggesting that ERVWE1 upregulated HTR1B expression by altering the m6A modification level (Fig. 6H). Moreover, ALKBH5 facilitated HTR1B expression, but depletion of ALKBH5 by RNA interference led to a decline in HTR1B protein levels in SH-SY5Y cells (Additional file 1: Fig. S8C-E). To explore whether ALKBH5 directly bound to HTR1B transcripts, we observed a significant increase in the enrichment of ALKBH5 in HTR1B mRNA in the presence of ERVWE1 using RIP-qPCR (Fig. 6I), demonstrating that ERVWE1-regulated HTR1B mRNA m6A modification was mediated by ALKBH5. Furthermore, the loss of ALKBH5 significantly attenuated the ERVWE1-induced upregulation of HTR1B and downstream proteins p-ERK1/2, p-ELK1, and Arc (Fig. 6J and Additional file 1: Fig. S8F), indicating the participation of ALKBH5 in the upregulation of HTR1B by ERVWE1. In summary, ERVWE1 altered HTR1B expression by regulating ALKBH5-mediated m6A demethylation.

### ERVWE1 increased HTR1B expression by maintaining its mRNA stability through ALKBH5-induced m6A demethylation

**Fig. 7:**
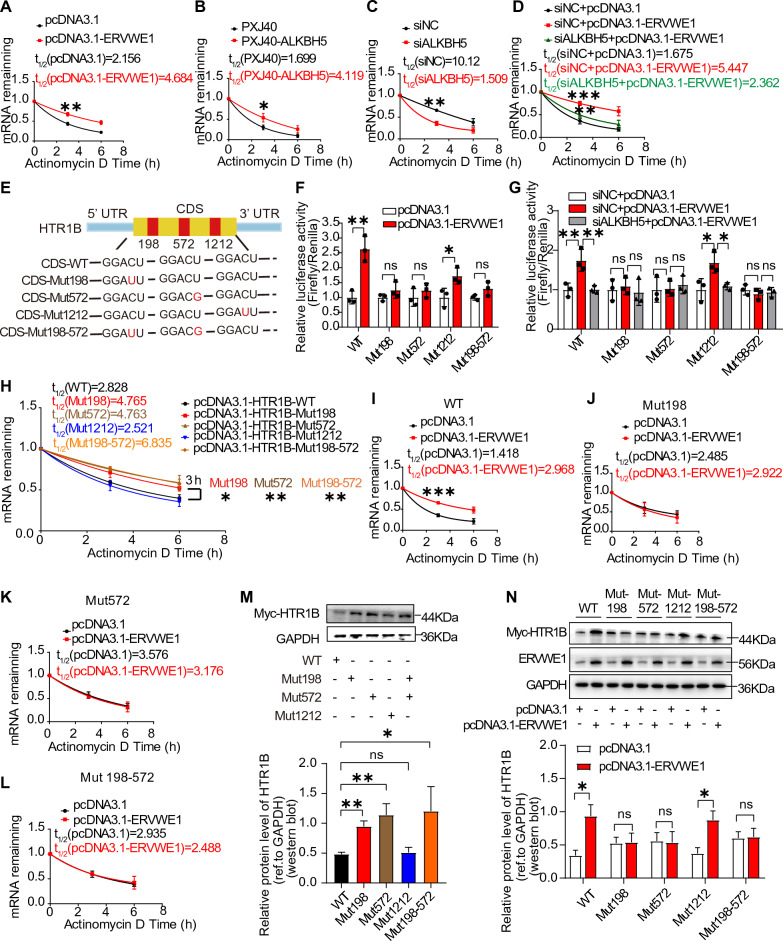
ERVWE1 upregulated HTR1B via enhancing its mRNA stability. **A**–**D** The mRNA half-life (t_1/2_) of HTR1B in SH-SY5Y cells after transfection with **A** ERVWE1, **B** ALKBH5, **C** siALKBH5 or siCtrl, and co-transfection with **D** ERVWE1 (or control) and siALKBH5(or siNC) by Actinomycin D (5 µg/mL) treatment. Cells were harvested at 0, 3, and 6 h. The expression level was normalized to that at “0 h”, and GAPDH was used as a control. The mRNA decay rate was analyzed by nonlinear regression curve fitting (one-phase decay model). **E** Putative m6A modification sites in the coding sequence of HTR1B and synonymous mutations. **F**, **G** Relative luciferase activity of HTR1B with either wild-type or mutant m6A sites after co-transfection with ERVWE1 or control vector (**F)** and tri-transfection with ERVWE1 (or control) and siALKBH5 (or siNC) vectors (**G**) in SH-SY5Y cells. **H** The decay of HTR1B mRNA in SH-SY5Y cells with CDS-WT and CDS-mutant. **I**–**L** Decay of HTR1B mRNA with ERVWE1 overexpression versus control in the HTR1B-CDS-WT and mutant m6A sites groups in SH-SY5Y cells. **M** The protein level of HTR1B after mutation of m6A sites in SH-SY5Y cells. **N** The protein level of HTR1B between pcDNA3.1 and pcDNA3.1-ERVWE1 group after transfected with mutation plasmids in the SH-SY5Y. Data are mean ± SD. Statistical analysis: Student’s *t*-test or one-way ANOVA (^ns^ *p* > 0.05, *p < 0.05, **p < 0.01, ***p < 0.001). All experiments were repeated 3 times

M6A modification plays a role in regulating messenger RNA stability [60]. To investigate whether ERVWE1 enhances HTR1B expression by regulating its mRNA stability, we assessed the mRNA half-life of HTR1B under Actinomycin D treatment, which blocks de novo RNA synthesis. ERVWE1 significantly increased the stability of HTR1B mRNA (Fig. 7A). Similarly, ALKBH5 augmentation prolonged the stability of HTR1B mRNA, whereas depletion of ALKBH5 resulted in a distinct decline in stability (Fig. 7B, C). Strikingly, interfering with ALKBH5 abolished the ERVWE1-induced enhancement of HTR1B mRNA stability (Fig. 7D), indicating that ERVWE1 influenced HTR1B mRNA stability through ALKBH5. Analysis using the SRAMP website (http://www.cuilab.cn/sramp) and RMBase v2.0 (https://rna.sysu.edu.cn/rmbase/index.php) revealed the presence of several high confidence predicted or empirical m6A modification, primarily located in the CDS (coding sequences) (Additional file 1: Fig. S9A, B). We selected the three highest confidence m6A sites (198, 572, 1212) distributed across the sequences and mutated those sites, termed CDS-Mut198, CDS-Mut572, CDS-Mut1212, and CDS-Mut198-572 (Fig. 7E). Subsequently, we conducted a reporter luciferase assay and observed that ALKBH5 enhanced luciferase activity in both HTR1B CDS wild type (WT) and Mut1212, but had no effect on Mut198, Mut572, and Mut198-572 (Additional file 1: Fig. S10A, B). Moreover, ERVWE1 increased luciferase activity in the HTR1B WT and Mut1212, but not in Mut198, Mut572, and Mut198-572 (Fig. 7F). Intriguingly, ALKBH5 silencing almost completely abolished the ERVWE1-induced luciferase activity in HTR1B CDS WT and Mut1212 groups, while the luciferase activity showed no change in Mut198 and Mut572 groups, suggesting potential methylation modified sites at positions 198 and 572 (Fig. 7G). This finding was further confirmed by mutation of both 198 and 572 sites (Fig. 7G). Taken together, sites 198 and 572 are likely functional m6A-modified sites in the CDS regions of HTR1B.

Next, we assessed the half-life of HTR1B mRNA and observed that mutation of sites 198 or 572 sites, but not 1212 site, prolonged the half-life of HTR1B mRNA transcripts compared to WT in SH-SY5Y cells (Fig. 7H), indicating that 198 and 572 sites were the m6A modification motifs in HTR1B mRNA. Mutation of both sites 198 and 572 further validated these results (Fig. 7H). ERVWE1 indeed perpetuated HTR1B mRNA stability in CDS WT groups (Fig. 7I). However, there were no changes in the Mut198 or Mut572 groups (Fig. 7J, K). ERVWE1 lengthened the half-life of HTR1B in the Mut1212 group (Additional file 1: Fig. S10C), demonstrating that ERVWE1 enhanced HTR1B stability dependent on two sites 198 and 572. This finding was also confirmed in Mut198-572 groups (Fig. 7L). To further support the result, we measured the protein expression levels of HTR1B in SH-SY5Y cells and discovered that mutations in the site 198, 572, or both 198 and 572 could reinforce HTR1B expression compared to WT-types (Fig. 7M). In CDS WT and Mut1212 cells, ERVWE1 significantly augmented HTR1B protein levels (Fig. 7N and Additional file 1: Fig. S10D). There was no difference in HTR1B expression in Mut198, Mut572, or Mut198-572 (Fig. 7N). These results suggested that ERVWE1 regulated HTR1B mRNA stability via the m6A-modified sites 198 and 572. In conclusion, ERVWE1 upregulated HTR1B expression by increasing its mRNA stability dependent on 198 and 572 sites.

### ALKBH5 participated in the regulation of synaptic plasticity by ERVWE1 in 5-HT neurons

**Fig. 8:**
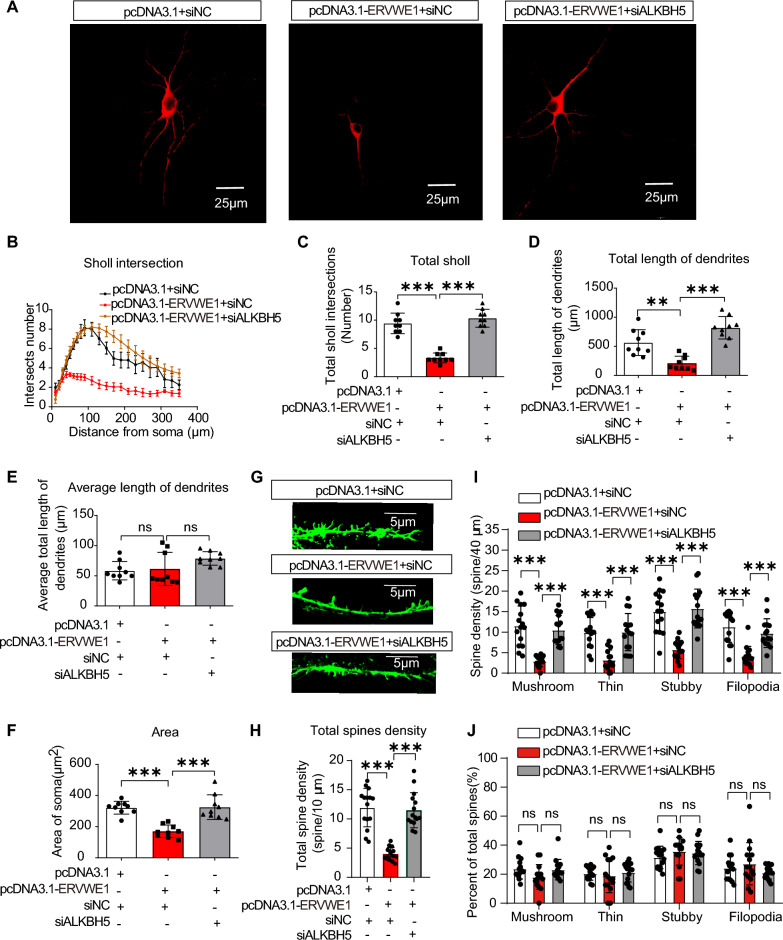
ERVWE1-influenced 5-HT neuronal plasticity was mediated by ALKBH5. **A**-**F** The complexity of 5-HT neurons in the ERVWE1-expressed and empty plasmid groups after co-transfection with ALKBH5 deficiency or control vectors by Sholl analysis. Scale bars = 25 μm. **A** 5-HT neuron morphology. **B**, **C** Sholl intersection numbers (*n* = 9). **D** Total branching length (*n* = 9). **E** The average length of branching (*n* = 9). **F** The area of soma (*n* = 9). **G**–**J** The spine density of 5-HT neurons in pcDNA3.1 + siNC, pcDNA3.1-ERVWE1 + siNC, pcDNA3.1-ERVWE1 + siALKBH5 groups. **G** The diameter of dendrites of 5-HT neurons. Scale bars = 5 μm. **H** Total spine density (*n* = 15). **I** Each subtype’s spine density (*n* = 15). **J** The percentage of spine density in four types (*n* = 15). Data are mean ± SD. Statistical analysis: one-way ANOVA (^ns^ *p* > 0.05, **p < 0.01, ***p < 0.001)

Based on the significant impact of ALKBH5 demethylation on ERVWE1-induced HTR1B upregulation, we investigated whether ERVWE1-impaired synaptic plasticity was mediated by ALKBH5. Immunofluorescence results showed that the increased immunoreactivity of Arc induced by ERVWE1 was reduced when cells were treated with siALKBH5 oligos compared to siRNA controls (Additional file 1: Fig. S11A, B). Furthermore, we analyzed the morphology of 5-HT dendrites and observed that ERVWE1 decreased the number of dendritic branches (Fig. 8A–C) and the total length of dendrites (Fig. 8D), but not the average dendritic length (Fig. 8E). However, knockdown of ALKBH5 reversed the decrease in 5-HT neuronal complexity induced by ERVWE1 (Fig. 8A–E). In addition, ALKBH5 also regulated the soma size of 5-HT neurons (Fig. 8F). These results indicated that ALKBH5 influenced the complexity of 5-HT neurons regulated by ERVWE1.

Moreover, we observed that ERVWE1 significantly decreased the total spine density in 5-HT neurons, while the total spine density returned to normal levels after knocking down ALKBH5 in ERVWE1 overexpressed cells (Fig. 8G, H). Notably, both mature spines (mushroom or stubby) and immature spines (thin or filopodia) were regulated by ALKBH5 compared with the control groups (Fig. 8I). In contrast, there were no significant differences in the proportion of these four subtypes (Fig. 8J), indicating that ERVWE1-regulated 5-HT neuronal plasticity was mediated by ALKBH5. In summary, ERVWE1 impaired synaptic plasticity in 5-HT neurons in an ALKBH5-m6A-dependent manner.

## Discussion

The etiology of schizophrenia is complex. Our lab’s data have demonstrated that ERVWE1 is the underlying pathogenic factor responsible for schizophrenia [35]. Further studies indicate that ERVWE1 increases the expression of schizophrenia risk genes, such as Brain derived neurotrophic factor (BDNF) [35] and Cytoplasmic polyadenylation element binding protein 1(CPEB1) [36]. An in-depth study finds that ERVWE1 upregulates BDNF expression by increasing the phosphorylation of Glycogen synthase kinase 3β (GSK3β) at Ser9 [61]. Moreover, ERVWE1 activates the TRPC3 channel, leading to an influx of Ca^2+^ [62] by inhibiting Disrupted in schizophrenia 1 (DISC1) and subsequently triggering the activation of Small-conductance calcium-activated potassium type 2 channel (SK2) [18] and type 3 channel (SK3) [63]. ERVWE1 can also inhibit complex I activity, thereby impacting the mitochondrial respiratory chain in schizophrenia [36]. Intensive analysis indicates that ERVWE1 induces human leukemia antigen-A*0201-restricted cytotoxic T lymphocytes [42] and promotes neuroinflammation through increasing NO production and enhancing microglial migration [64]. Additionally, ERVWE1 interacts with toll-like receptors 3 or 4, which are pattern recognition receptors, thereby increasing interleukin-6 (IL-6) and C-reactive protein (CRP), or IL-10 and tumor necrosis factor-α (TNF-α), and ultimately inducing an innate immune response [40, 65]. ERVWE1 also triggers an antiviral innate immune response and causes neuronal apoptosis by increasing Interferon beta (IFN-β) expression via the linc01930/cGAS/STING pathway [39]. Recently, we have shown that ERVWE1 triggers hyperactivity in dopaminergic neurons via mediating dopamine synthesis, reuptake, and transport and enhancing sodium influx [38]. Besides, ERVWE1 affects hippocampal neuron dendritic morphology and decreases spine density by inhibiting the Wnt/JNK non-canonical pathway [37]. In brief, ERVWE1 is a significant risk factor for schizophrenia. In this paper, we demonstrated that ALKBH5 and HTR1B were increased in first-episode schizophrenia and displayed a strong correlation with ERVWE1. ALKBH5 was a new risk gene for schizophrenia, and it is significantly related to HTR1B in schizophrenia patients. Further studies established that ERVWE1 impaired 5-HT plasticity through the HTR1B signal pathway in an ALKBH5-m6A-dependent manner in schizophrenia.

The GSE53987 samples are obtained from the postmortem prefrontal cortex of individuals with schizophrenia and healthy controls. This brain region exhibits a significant differential expression of genes in schizophrenia [66, 67]. Lanz and his colleagues identify inflammatory abnormalities in schizophrenia using the GSE53987 [66]. However, our bioinformatics analysis demonstrated altered expression of the 5-HT systems in the PFC of individuals with schizophrenia. We observed an increase in HTR1B mRNA expression in GSE53987, which is consistent with the findings of Lopez-Figueroa [6]. Additionally, our results also revealed a decrease in HTR2A expression in the PFC of individuals with schizophrenia, which is in line with a meta-analysis of postmortem study [6]. Additionally, our bioinformatics analysis did not find a significant difference in HTR1A expression between individuals with schizophrenia and healthy controls. However, Lopez-Figueroa and Selvaraj observe an alteration in HTR1A mRNA levels in the prefrontal cortex of individuals with schizophrenia [6, 68]. The inconsistent results can be attributed to factors such as sample size and exposure to antipsychotic medications.

The heterogeneity and complexity of schizophrenia make diagnosis challenging. Consequently, blood biomarkers have emerged as a promising tool for aiding in diagnosis and predicting clinical outcomes in schizophrenia due to significant advancements in proteomics [69]. A recent study reports that the level of N-methyl-D-aspartate (NMDA) glutamate receptor [70], dopamine receptors (DRD1-4), and HTR3 may potentially serve as peripheral biomarkers for schizophrenia [17]. In our study, we reported a higher level of HTR1B in the blood of first-episode schizophrenia patients compared to healthy controls, suggesting that HTR1B may serve as a potential new blood biomarker for schizophrenia. However, we did not observe any differences in the mRNA expression of HTR1A and HTR2A in schizophrenia, consistent with the findings of Wysokinski and colleagues [17]. Furthermore, one study demonstrates that elevated HTR1A expression is found in blood leukocytes of male antipsychotic-free patients with schizophrenia, whereas decreased HTR1A level is observed in female antipsychotic-free patients [71], suggesting that gender may be a crucial factor influencing HTR1A expression in acute schizophrenic patients. Moreover, we observed increased expression of the plasticity-related gene Arc in first-episode schizophrenia patients. Previous studies have reported alterations in the expression of plasticity-associated genes such as BDNF, NGF, VEGF, and NRG1 in the blood of individuals with schizophrenia [7, 72], which may provide new evidence for synaptic plasticity dysfunction in schizophrenia. Aberrant expressions of METTL3 and FTO have been associated with Alzheimer’s or Parkinson’s disease [51]. However, there is no study reporting the role of m6A modification in schizophrenia to date. Here, we reported that ALKBH5, an important demethylase of m6A, was significantly increased in the blood of schizophrenia. Importantly, further analysis revealed that ALKBH5 was an independent risk factor for schizophrenia. Previous study reports that ALKBH5 of peripheral blood immune cells is a prospective biomarker for the diagnosis of non-small cell lung cancer [73]and targeting ALKBH5 may be a promising therapeutic method for gastric cancer patients [74]. Additionally, ALK-04, a small-molecule ALKBH5 inhibitor, enhanced the efficacy of cancer immunotherapy [75]. Therefore, we proposed that ALKBH5 and HTR1B may serve as potential blood biomarkers for diagnosing and individualizing pharmacotherapy for patients with schizophrenia, highlighting a novel avenue for therapeutic targeting.

Since our clinical data indicated a strong positive correlation between HTR1B and ERVWE1, we conducted a series of in *vitro* experiments to examine the regulation of HTR1B expression by ERVWE1. HTR1B, located on the presynaptic and postsynaptic terminals of serotonergic neurons, couples through G (ialpha2), leading to a decrease in cellular cAMP (Cyclic adenosine monophosphate) levels [9]. It also activates the ERK signal pathway, which plays a role in regulating synaptic plasticity in the postsynaptic membrane [8–10]. The canonical ERK signaling pathway involves the activation of Ras-Raf-MEK-ERK-ELK1 kinases through cascade phosphorylation of downstream proteins, and it appears to be important in neurodevelopment [76]. Both hypoactivation and hyperactivation of Ras signaling can impair synaptic plasticity [77]. Deletion of MEK1/2 leads to reduced expression of Arc [78]. A recent study reveals that an ERK1/2 inhibitor blocks the upregulation of BDNF induced by MK-801, which mimics schizophrenia-like symptoms in healthy individuals [79], suggesting that ERK1/2 plays a critical role in schizophrenia. In this article, we reported that ERVWE1 amplified the expression of HTR1B, p-ERK1/2, p-ELK1, and Arc in SH-SY5Y cells and 5-HT neurons, indicating that ERVWE1 could upregulate Arc expression through the ERK cascade, thus potentially participating in the development of schizophrenia.

De novo synthesized Arc mRNA can be rapidly transported to dendrites and enriched in the postsynaptic density [80]. Arc can interact with synaptic proteins, including AMPAR, CaMKIIβ, or presenilin 1, which are essential components in the formation of stable synaptic plasticity [81, 82]. Dysfunction in synaptic plasticity includes abnormalities in synaptic plasticity-associated genes, dendrite morphology, and spine density. The complexity of neurons (including branching numbers and length), which is associated with synapse formation and loss, has been implicated in the etiology of schizophrenia [83]. Our results demonstrated that ERVWE1 profoundly affected the complexity of 5-HT neurons, resulting in a significant decrease in dendrite length and branching number. Increasing evidence suggests that dendrite morphological abnormalities contribute to schizophrenia [84, 85]. Studies using postmortem human tissue have indicated a decrease in soma size rather than neuron loss in the PFC of schizophrenia [86]. Interestingly, we found that ERVWE1 reduced the soma size of 5-HT neurons. Additionally, a decrease in spine density has been observed in schizophrenia subjects in multiple brain regions including the frontal and temporal neocortex [16] and the dorsolateral prefrontal cortex of patients with schizophrenia [87]. The prevailing hypothesis of reduced spine density is that it increases the pruning of existing synapses, particularly large or mature spines. However, a recent study has suggested a loss of small spines in schizophrenia [88]. Our results revealed that ERVWE1 reduced spine density not only in mature spines (mushroom and stubby) but also in immature spines (thin and filopodia). Additionally, our previous study has shown that ERVWE1 can reduce spine density in hippocampal neurons by inhibiting the Wnt/JNK non-canonical pathway in schizophrenia, leading to abnormalities in neuronal development [37]. Spine development is regulated by multiple factors and is a complex biological process. It is possible that ERVWE1 may also negatively impact DRN spine development via the Wnt/JNK non-canonical pathway. All of these findings likely hold an important key to understanding ERVWE1’s role in pathogenesis, as synaptic plasticity impairments are a leading cause of cognitive dysfunction in schizophrenia.

Our results suggested that HTR1B was involved in the expression of Arc induced by ERVWE1. Arc plays a role in impaired synaptic plasticity by regulating AMPAR endocytosis [58] or disperses AMPA receptors by modulating the postsynaptic density (PSD) phase separation [14]. We also observed that HTR1B was involved in the impaired plasticity of 5-HT neurons induced by ERVWE1. The HTR1B agonist CP93129 has been shown to significantly decrease the spine density in CA1 pyramidal neurons [89]. Intriguingly, data from our lab has demonstrated that ERVWE1 activates the SK2 channel by inhibiting HTR4, which is involved in the development of schizophrenia [18]. Furthermore, the HTR4 agonist RS67333 significantly promotes synaptic plasticity, including an increase in total dendritic length, the number of primary dendrites, and dendritic branching [90]. Although both HTR1B and HTR4 are 5-HT receptors, they belong to different subfamilies and have distinct functions. Therefore, we proposed that ERVWE1 impaired the plasticity of 5-HT neurons by activating the HTR1B signaling pathway to regulate synaptic plasticity-associated gene expression in schizophrenia.

A series of studies suggest that epigenetic modifications, such as histone modifications, DNA methylation, or RNA methylation, regulate ERVs and control ERVs elements silently [22, 91]. However, as is known to us, only two articles have reported the impact of ERVs on epigenetic modifications. One study shows that ERV-derived enhancers increase H3K27ac and reduce H3K9me3, thereby controlling the transcriptional program of trophoblast syncytialization [92]. Another study reports that HERV-H-derived lncRNAs regulate H3K27ac modification [93]. Nevertheless, there is no literature reporting that HERV proteins contribute to epigenetic modifications, particularly RNA modification. M6A modification is a primary RNA epigenetic regulatory mechanism that controls gene expression in eukaryotic mRNA [94]. Infection with exogenous viruses, such as Dengue virus, Zika virus, West Nile virus, Hepatitis C virus, and SARS-CoV-2 variants, can alter the m6A modification of host mRNAs [95, 96]. Additionally, virus proteins Hepatitis B virus X [97] and HIV-1 envelope [98] regulate m6A levels of cellular RNA. Here, we first reported that the endogenous viral protein, ERVWE1, reduced the global m6A levels of cellular RNA. M6A modification requires the coordination of “writers”, “erasers”, and “reader” proteins in those processes [99]. We discovered that ERVWE1 could enhance ALKBH5 promoter activity, thereby increasing the expression of the m6A demethylase ALKBH5. Furthermore, our previous study indicates that ERVWE1 interacts with transcription factors (TFs) such as Yin Yang 1 (YY1) [37]. It is possible that ERVWE1 facilitates TF binding to the ALKBH5 promoter, ultimately contributing to ALKBH5 upregulation. Additionally, ALKBH5 was involved in ERVWE1 mediated-m6A modification. Knockdown of ALKBH5 could counteract the decrease in global m6A levels regulated by ERVWE1. Our study provides the first evidence that HERVs could regulate m6A modification suggesting that ERVWE1 has the potential to affect the m6A modification of host cell transcripts.

In addition, our bioinformatics and clinical results showed a strong positive correlation between HTR1B and ALKBH5. Further studies demonstrated that HTR1B was a direct target of m6A modification and ALKBH5 was involved in ERVWE1-induced upregulation of HTR1B by removing m6A modification. ALKBH5 functions as an RNA m6A demethylase [100]. Recent research has shown that ALKBH5 impacts mRNA stability and expression [101]. Similarly, we found that ERVWE1 enhanced HTR1B mRNA stability by reducing its mRNA m6A level through ALKBH5. Most studies have revealed that ALKBH5 enhances the mRNA stability of target genes [102, 103], particularly at m6A-modified sites within the translated regions (coding sequence) [104]. Our results demonstrated that ERVWE1 enhanced HTR1B stability and expression through an ALKBH5-m6A methylation-dependent mechanism within the HTR1B coding sequence. However, few studies have reported a negative correlation between ALKBH5 and mRNA stability when the m6A-modified sites are near stop codons [105], as the m6A sites in the 3′-UTRs (3′-untranslated region) generally lead to decreased mRNA stability. We first identified that ALKBH5 influenced mRNA stability in schizophrenia.

The widespread distribution of m6A modification throughout the mammalian brain highlights its importance as a post-transcriptional regulator in the nervous system. It plays a role in the regulation of various neuronal activities and functions, including spine outgrowth, neurite extension, axon guidance, and synapse formation [106]. Reduced levels of m6A modification can negatively impact synaptic function [107], leading to impaired synaptic translation of Glua1 mRNAs, and weakened neuronal activity. ALKBH5 is present at active synaptic ribosomes and synapses during short-term plasticity, indicating its involvement in synaptic maturation and providing evidence for its role in synaptic plasticity [108]. However, the precise influence of ALKBH5 on synaptic plasticity needs to be better understood. Our study suggested that ALKBH5 participated in the regulation of 5-HT complexity and spine density modulated by ERVWE1. Additionally, selective knockdown of YTHDC1 and YTHDF3 results in impairments in immature spine morphology and dampened excitatory synaptic transmission [109], highlighting the roles of m6A on synaptic plasticity. Taken together, ERVWE1 impaired neuronal plasticity in schizophrenia by activating the HTR1B signaling pathway in an m6A-dependent manner, and ALKBH5 was involved in these processes.

Although progress has been made in the potential etiology of schizophrenia, the prospect of treatment of schizophrenia has not kept up. Most antipsychotics target the receptors or transporters of dopamine, glutamate, and serotonin, but they have side effects, and their effectiveness in treating cognitive symptoms in schizophrenia patients is limited [110]. Immunotherapy is a hot field in the research of schizophrenia. To our knowledge, monoclonal antibodies immunotherapy targeted human IFN-γ-1b [111] and IL-6R [112] in schizophrenia are ongoing, particularly IL-6R monoclonal antibody has significant improvements in cognition. The effects of monoclonal antibody therapy are different, so it is necessary to identify biological targets with schizophrenia risk genes. Both this paper and our previous research show that ERVWE1 is higher in patients with schizophrenia, and it is a risk gene for schizophrenia [18, 35–40, 113]. GNbAC1 is a humanized IgG4 monoclonal antibody designed to antagonize the surface domain of the ERVWE1 protein [114]. It has been reported that GNbAC1 could be used to treat MS patients. Nowadays, GNbAC1 is being tested in a phase II trial. Additionally, GNbAC1 also develops as a new therapeutic approach for type 1 diabetes [115]. Therefore, we proposed that a monoclonal antibody targeting ERVWE1 might be a novel therapy for schizophrenia patients.

## Conclusions

**Fig. 9:**
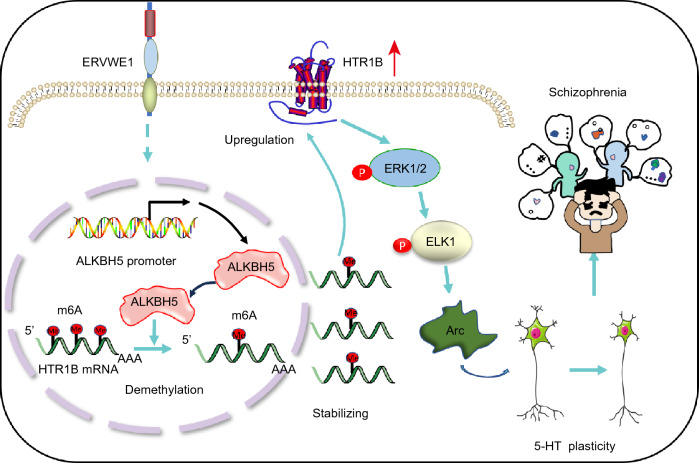
A possible hypothesis that ERVWE1 impaired 5-HT neuronal plasticity by posttranscriptional activation of HTR1B in ALKBH5-m6A dependent epigenetic mechanisms in schizophrenia. ERVWE1 reduced the global m6A level depending on ALKBH5 demethylation, contributing to HTR1B upregulation and activating the downstream signaling pathway, which led to a decrease in 5-HT neuronal dendrite complexity and spine density in schizophrenia

We observed higher levels of HTR1B, Arc, and ALKBH5 in individuals with schizophrenia. Importantly, HTR1B, Arc, and ALKBH5 had a strong positive correlation with ERVWE1 in schizophrenia patients. ALKBH5 may be an independent risk factor for schizophrenia. In *vitro* experiments indicated that ERVWE1 increased ALKBH5 expression by enhancing its promoter activity, which subsequently reduced the m6A modification of HTR1B mRNA. The decreased m6A level of HTR1B enhanced the stability of HTR1B mRNA, resulting in HTR1B upregulation and activating the ERK1/2-ELK1-Arc signal pathway. This activation led to impairments in 5-HT neuronal plasticity. This finding may provide a novel cellular and molecular mechanism for the involvement of ERVWE1 in the etiology of schizophrenia and open new possibilities for drug development targeting ERVWE1 and ALKBH5 to treat schizophrenia patients (Fig. 9).

## Materials and methods

### Blood samples

Blood samples from first-episode schizophrenia patients (including 15 whole peripheral blood samples and 44 plasma samples) and healthy controls (including 14 whole peripheral blood samples and 37 plasma samples) were recruited from Wuhan Mental Health Center. Consent/permission had been obtained from the participants. There were no significant differences in age, education, body mass index (BMI), gender, and smoking status between schizophrenia patients and healthy individuals (Additional file 1: Tables S5, S6). We used the Diagnostic and Statistical Manual of Mental Disorders (Fifth Edition) to diagnose patients with schizophrenia. Exclusion criteria were comorbid psychiatric diagnoses, acute infectious, or inflammatory. Additionally, healthy controls should not have any neurological or psychiatric diseases. We collected blood samples using Ethylenediaminetetraacetic acid (EDTA)-coated tubes and centrifuged at 3,000 r/min for 10 min to obtain plasma for ELISA. Meanwhile, we added TRIzol LS reagent (Invitrogen, 10296010, USA) to whole blood to isolate total RNA for RT-qPCR. All blood samples were stored at − 80 ℃ until analysis.

### Statistical analysis

Median and nonparametric analyses were used for the analysis of clinical results by SPSS 20. Correlation analysis was performed using online software (http://sangerbox.com/), and *p* values were analyzed by Spearman. One-way analysis of variance (ANOVA) and student’s *t*-test were used for statistical analyses using GraphPad Prism 8.0. A *p* < 0.05 was considered statistically significant. Data were shown as mean ± SD (standard deviation). All experiments were repeated more than 3 times.

### Supplementary Information

**Additional file 1: Table S1.** The concentration of SERT in the plasma of healthy controls and schizophrenia patients. **Table S2.** The concentration of TPH2 in the plasma of healthy controls and schizophrenia patients. **Table S3.** The concentration of 5-HT in the plasma of healthy controls and schizophrenia patients. **Table S4.** The concentration of ERVWE1 in the plasma of healthy controls and schizophrenia patients. **Table S5.** Comparison of the whole peripheral blood samples demographic data between the healthy controls and recent-onset schizophrenia patients. **Table S6.** Comparison of the plasma samples demographic data between the healthy controls and recent-onset schizophrenia patients. **Table S7.** Primer sequences used in real-time quantitative PCR (RT-qPCR). **Table S8.** Primer sequences used in plasmid constructs and siRNA. **Table S9. **Antibodies used in western blot. **Fig S1.** 5-HT receptors abnormality in the prefrontal cortex (BA46) of schizophrenia by GSE53987. A-F Boxplot of 5-HT receptors expression in schizophrenia (*n* = 15) vs healthy controls (*n* = 19), A Boxplot of HTR2A expression, B Boxplot of HTR1A expression, C Boxplot of HTR2B expression, D Boxplot of HTR5A expression, E Boxplot of HTR6 expression, F Boxplot of HTR7 expression. *p*-valve was analyzed by wilcoxon (Mann-Withney). Data shown are the mean ± SD. **Fig S2.** mRNA expression level of 5-HTergic systems, Arc, and ALKBH5 in the whole peripheral blood of schizophrenia patients and healthy controls. A-I Respectively represent the mRNA expression levels of HTR1B, HTR1A, HTR2A, HTR6, HTR7, SERT, TPH2, Arc, and ALKBH5 in the whole peripheral blood of schizophrenia patients (*n* = 15) and healthy controls (*n* = 14) by RT-qPCR (*p*-value by median and nonparametric analysis). J-L Correlation of ERVWE1 mRNA level with HTR1B (*p* = 0.01, r = 0.65), Arc (*p* = 0.03, r = 0.57), and ALKBH5 (*p* < 0.01, r = 0.78) mRNA levels in schizophrenia by Spearman. Dots depict schizophrenia patients, but a few are overlapping and cannot be separated on the graph. Data shown are the mean ± SD. **Fig S3**. ERVWE1 overexpressed in the SH-SY5Ycells. A The representative western blot results of ERVWE1. B Protein expression level of ERVWE1. Data shown are the mean ± SD and represent three independent experiments. Statistical analysis: Student’s t-test (*p < 0.05). **Fig S4.** HTR1B activated ERK-ELK1-Arc signal pathway in the SH-SY5Y cells. A-D Representative western blot results for ERK1/2, p-ERK1/2, ELK1, p-ELK1, and Arc proteins in the SH-SY5Y cells after transfection with HTR1B expressed vectors and siRNA oligo respectively. Data shown are the mean ± SD and represent three independent experiments. Statistical analysis: Student’s t-test (ns *p* > 0.05, *p < 0.05, **p < 0.01). **Fig S5.** ERVWE1 activated HTR1B signal pathway in SH-SY5Y cells. The protein expression levels of ERVWE1 and HTR1B were detected in cells co-transfected with ERVWE1 and siHTR1B. Data shown are the mean ± SD and represent three independent experiments. Statistical analysis: one-way ANOVA (*p < 0.05, **p < 0.01). **Fig S6.** ERVWE1 activated HTR1B signal pathway in DRN neurons. A Transfection efficiency of ERVWE1 in DRN neurons by western blot. B Immunofluorescence staining with HTR1B in DRN neurons. C The western blot results of ERVWE1 and HTR1B were detected by applying siHTR1B and ERVWE1. Data shown are the mean ± SD and represent three independent experiments. Statistical analysis: Student’s t-test and one-way ANOVA (*p < 0.05, **p < 0.01). **Fig S7.** The transfection efficiency of ERVWE1 in the DRN neurons. Scale bars = 25 μm. **Fig S8.** ALKBH5 regulated m6A modification in SHSY5Y. A, B RNA dot blot analysis of m6A levels. A ALKBH5 expressed cells vs control cells, B siALKBH5 cells and siNC cells. Methylene blue staining served as a loading control. C-E Western blot results of ALKBH5 and HTR1B. C, D ALKBH5 overexpression, E ALKBH5 knockdown. F The western blot results of ERVWE1 and ALKBH5 were detected when ERVWE1 co-transfected with siALKBH5 or siNC control. Data shown are the mean ± SD and represent three independent experiments. Statistical analysis: Student’s t-test and one-way ANOVA (*p < 0.05, **p < 0.01, ***p < 0.001). **Fig S9.** m6A modification sites predictor of HTR1B mRNA. A SRAMP. High and very high confidences were considered as a candidate site. B RMBase v2.0. Score above 400 was considered as a candidate site. **Fig S10.** ERVWE1 reduced the m6A level of HTR1B by 198 and 574 sites. A Schematic illustration of HTR1B with either wild-type or mutant m6A sites luciferase activity plasmids constructs. B Relative luciferase activity of HTR1B-CDS wild-type and mutant m6A sites after co-transfection with ALKBH5 expressing plasmid or control plasmid in SH-SY5Y cells. C Decay of HTR1B mRNA with ERVWE1 overexpression versus control in the HTR1B-CDS-Mut1212 groups in SH-SY5Y cells. D The relative protein levels of ERVWE1 in SH-SY5Y cells. Data shown are the mean ± SD and represent three independent experiments. Statistical analysis: Student’s t-test (^ns^ *p* > 0.05, *p < 0.05, ***p < 0.001). **Fig S11.** ALKBH5 participated in ERVWE1-induced Arc upregulation in DRN neurons. A Immunofluorescence staining with Arc in DRN neurons. B The relative intensity of Arc. Data shown are the mean ± SD and represent three independent experiments. Statistical analysis: one-way ANOVA (**p < 0.01).

## Data Availability

All relevant data are available from the authors.

## Abbreviations

3′-UTRs: 3′-Untranslated regions
5-HT: 5-Hydroxytryptamine
ALKBH5: AlkB homolog 5, RNA demethylase
AMPAR: α-Amino-3-hydroxy-5-methyl-4-isoxazolepropionic acid receptor
ANOVA: One-way analysis of variance
Arc: Activity regulated cytoskeleton associated protein
BDNF: Brain derived neurotrophic factor
CaMKII: The Ca^2+^/calmodulin (CaM)-dependent protein kinase II
cAMP: Cyclic adenosine monophosphate
CDS: Coding sequences
cGAS: Cyclic GMP-AMP synthase
CPEB1: Cytoplasmic polyadenylation element binding protein 1
CRP: C-reactive protein
DEGs: Differentially expressed genes
DISC1: Disrupted in schizophrenia 1
DRD: Dopamine receptors
DRN: Dorsal raphe nucleus
EDTA: Ethylenediaminetetraacetic acid
ELK1: ETS transcription factor ELK1
ERK: Extracellular signal-regulated kinase
ERVWE1: Human endogenous retrovirus W family envelope
FTO: FTO alpha-ketoglutarate dependent dioxygenase
GOBP: Gene ontology biological process
GOCC: Gene ontology cell component
GPCR: G-protein-coupled receptor
GSK3β: Glycogen synthase kinase 3β
GWAS: Genome-wide association studies
HERVs: Human endogenous retroviruses
HTR: 5-HT receptor
HTR1B: 5-HT1B receptor
IFN-β/γ-1b: Interferon beta/gamma-1b
IGF2BP1/2/3: Insulin like growth factor 2 mRNA binding protein 1/2/3
IL-6/10: Interleukin-6/10
JNK: C-Jun N-terminal kinase
KEGG: Kyoto encyclopedia of genes and genomes
KIAA1429: Vir like m6A methyltransferase associated
lncRNA: Long non-coding RNA
LTRs: Long terminal repeats
m6A: N6-methyladenosine
MAP2: Microtubule associated protein 2
MEK: Mitogen-activated protein
METTL3/14/16: Methyltransferase 3/14/16
MS: Multiple sclerosis
MSRV: Multiple sclerosis associated retrovirus
NGF: Nerve growth factor
NMDA: N-methyl-D-aspartate
NO: Nitric oxide
NRG1: Neuregulin 1
PFC: Prefrontal cortex
PSD: Postsynaptic density
Raf: Zinc fingers and homeoboxes 2
Ras: Rat sarcoma
RBM15/15B: RNA binding motif protein 15/15B
SK: Small-conductance calcium-activated potassium
STING: Stimulator of interferon genes
TFs: Transcription factors
TNF-α: Tumor necrosis factor-α
TRPC3: Transient receptor potential canonical 3
VEGF: Vascular endothelial growth factor
WTAP: WT1 associated protein
YTHDC1/2: YTH N6-methyladenosine RNA binding protein C1/2
YTHDF1/2/3: YTH N6-methyladenosine RNA binding protein F1/2/3
YY1: Yin Yang 1
ZC3H13: Zinc finger CCCH-type containing 13
