# Supplementary material for: Captive ERVWE1 triggers impairment of 5-HT neuronal plasticity in the first-episode schizophrenia by post-transcriptional activation of HTR1B in ALKBH5-m6A dependent epigenetic mechanisms
Source: Cell Biosci. 2023 Nov 21;13:213. doi: 10.1186/s13578-023-01167-4 (PMC10664518; doi:10.1186/s13578-023-01167-4)
Supplement: Supplementary file 1 — Additional file 1: Table S1. The concentration of SERT in the plasma of healthy controls and schizophrenia patients. Table S2. The concentration of TPH2 in the plasma of healthy controls and schizophrenia patients. Table S3. The concentration of 5-HT in the plasma of healthy controls and schizophrenia patients. Table S4. The concentration of ERVWE1 in the plasma of healthy controls and schizophrenia patients. Table S5. Comparison of the whole peripheral blood samples demographic data between the healthy controls and recent-onset schizophrenia patients. Table S6. Comparison of the plasma samples demographic data between the healthy controls and recent-onset schizophrenia patients. Table S7. Primer sequences used in real-time quantitative PCR (RT-qPCR). Table S8. Primer sequences used in plasmid constructs and siRNA. Table S9. Antibodies used in western blot. Fig S1. 5-HT receptors abnormality in the prefrontal cortex (BA46) of schizophrenia by GSE53987. A-F Boxplot of 5-HT receptors expression in schizophrenia (n = 15) vs healthy controls (n = 19), A Boxplot of HTR2A expression, B Boxplot of HTR1A expression, C Boxplot of HTR2B expression, D Boxplot of HTR5A expression, E Boxplot of HTR6 expression, F Boxplot of HTR7 expression. p-valve was analyzed by wilcoxon (Mann-Withney). Data shown are the mean ± SD. Fig S2. mRNA expression level of 5-HTergic systems, Arc, and ALKBH5 in the whole peripheral blood of schizophrenia patients and healthy controls. A-I Respectively represent the mRNA expression levels of HTR1B, HTR1A, HTR2A, HTR6, HTR7, SERT, TPH2, Arc, and ALKBH5 in the whole peripheral blood of schizophrenia patients (n = 15) and healthy controls (n = 14) by RT-qPCR (p-value by median and nonparametric analysis). J-L Correlation of ERVWE1 mRNA level with HTR1B (p = 0.01, r = 0.65), Arc (p = 0.03, r = 0.57), and ALKBH5 (p < 0.01, r = 0.78) mRNA levels in schizophrenia by Spearman. Dots depict schizophrenia patients, but a few are overlapping and cannot be separated [file 13578_2023_1167_MOESM1_ESM.docx]

**Supplementary Information for**

**Captive ERVWE1 triggers impairment of 5-HT neuronal plasticity in the first-episode schizophrenia by post-transcriptional activation of HTR1B in ALKBH5-m6A dependent epigenetic mechanisms**

Xiulin Wu^1^, Lianzhong Liu^2^, Xing Xue^1^, Xuhang Li^1^, Kexin Zhao^1^, Jiahang Zhang^1^, Wenshi Li^1^, Wei Yao^1^, Shuang Ding^1^, Chen Jia^1^, Fan Zhu^1,3*^

^1^ State Key Laboratory of Virology, Department of Medical Microbiology, School of Basic Medical Sciences, Wuhan University, Wuhan, 430071, China.

^2^ Wuhan Mental Health Center, Wuhan, 430071, China.

^3^ Hubei Province Key Laboratory of Allergy & Immunology, Wuhan University, Wuhan, 430071, China.

*Correspondence: Fan Zhu, Email: [fanzhu@whu.edu.cn](mailto:fanzhu@whu.edu.cn). State Key Laboratory of Virology, Department of Medical Microbiology, School of Basic Medical Sciences, Wuhan University, Wuhan, 430071, China.

This file includes:

Supplementary Materials and Methods

Supplementary Tables

Supplementary Figures

**Supplementary Materials and Methods**

**Bioinformatics analysis**

The gene expression data of GSE53987 were acquired from the National Center for Biotechnology Information (NCBI) Gene Expression Omnibus (GEO) datasets (<http://www.ncbi.nlm.nih.gov/geo/>). The GSE53987 dataset contained 15 schizophrenia samples and 19 healthy controls from the postmortem prefrontal cortex (BA46). The data were based on the GPL570 [HG-U133_ Plus_2] Affymetrix Human Genome U133 Plus 2.0 Array platform and the differentially expressed gene (DEG) analysis was performed using the limma package. We conducted the Gene Ontology (GO) and Kyoto Encyclopedia of Genes and Genomes (KEGG) analyses using online tools (<http://sangerbox.com/>) (*p* < 0.05).

**Real-time quantitative PCR**

Total RNA was isolated using TRIzol reagent (Invitrogen, 15596026, USA). The cDNA was subsequently synthesized using the ReverTra Ace qPCR RT Master Mix with gDNA Remover kit according to the manufacturer's instructions (Toyobo, FSQ-301, Japan). The qPCR amplification was performed in a Mini Opticon Detector (Bio-Rad, USA) using SYBR Green qPCR Master Mix (Invitrogen, K0251, USA), and the amplification program was as follows: 94℃ for 3 min, followed by 40 cycles at 94℃ for 10 s, 58℃ for 15 s, and 72℃ for 20 s. The previously described primers and other specific primers in this paper were designed by primers 3 plus (Table S7). GAPDH was used as a housekeeping gene, and the relative mRNA expression levels were calculated using the 2^-ΔΔCt^ method.

**ELISA**

We collected plasma samples from each individual and diluted them 8-fold before measuring. We used enzyme-linked immunosorbent assay (ELISA) kits to quantify the concentrations of ERVWE1 (MEIMIAN, MM-61248H1, China), HTR1B (Warner Bio, CK-E15327, China), ALKBH5 (MEIMIAN, MM-51947H1, China), Arc (MEIMIAN, MM-61648H1, China), 5-HT (Warner Bio, CK-E10030, China), SERT (Warner Bio, CK-E10031, China), and TPH2 (Warner Bio, CK-E15437, China) according to the manufacturer’s protocols.

**Plasmids and siRNA**

The plasmid pcDNA3.1-ERVWE1 was obtained as previously described [1]. ERVWE1 was cloned into the pEGFP-N1 (named ERVWE1-EGFP) to analyze the transfection efficiency in the DRN neurons. The wild-type coding sequence (CDS) fragment of HTR1B was amplified and then cloned into vectors pcDNA3.1(-) and pmirGLO for stability and luciferase reporter assays separately (named as pcDNA3.1-HTR1B/ pcDNA3.1-HTR1B-WT and pmirGLO-HTR1B-WT). Site-directed mutagenesis of the plasmids encoding HTR1B (Mut 198, Mut572, Mut1212, and Mut 198-572) were generated using the Q5 Site-Directed Mutagenesis Kit (NEB, E0554, China). The plasmid PXJ40-ALKBH5 was constructed by inserting the ALKBH5 gene fragment into *Hind*III and *Kpn*I sites. The ALKBH5 promoter region (chr17:18182945-18184444) was cloned into the pGL3-basic vector using *Kpn*I and *Hind*III restriction enzymes to study the effect of ERVWE1 on ALKBH5 promoter activity. All primers for the plasmid constructs were listed in Table S8). Additionally, the small interfering RNA (siRNA) of HTR1B and ALKBH5 were synthesized by Sangon (Sangon Biotechnology Co., Ltd, Shanghai, China), and the sequences were listed in Table S8.

**Cell culture and transfection**

The human neuroblastoma cell line SH-SY5Y was purchased from the ATCC and grown in a mixture of Minimum Essential Media (Gibco, 61100061, USA) and F12 Medium (Gibco, 21700075, USA) supplemented with 10% FBS (Gibco, 10100147, USA), 1% penicillin-streptomycin (Gibco, 15140163, USA) and 100 mmol/L sodium pyruvate (Gibco, 11360070, USA) in a humidified atmosphere of 5% CO_2_ and 95% air at 37°C. Cells were transfected with Lipofectamine™ 2000 (Gibco, 11668500, USA) according to the manufacturer’s instructions.

**Western blot**

SH-SY5Y cells and primary cells were lysed using M-PER (Thermo, 78505, USA) containing a protease inhibitor cocktail (Roche Life Science, 4693159001, USA) at 48 h post-transfection. A BCA assay was used to quantify protein concentration. 25 μg of total protein was resolved by 10% SDS-PAGE and transferred onto a PVDF membrane (Millipore, PVH00010, USA). Afterwards, the PVDF membrane was blocked by 5% non-fat milk and incubated with the primary antibody overnight at 4℃. After washing with TBST, the membranes were incubated with secondary antibodies and developed using enhanced chemiluminescence (Millipore, WBKLS0100, USA). All antibodies used in this study were listed in Table S9.

**Isolation and culturing of 5-HT neurons**

The 5-HT neurons mainly originate in the dorsal raphe nucleus (DRN). Thus, we dissected and dissociated the DRN from the brains of newborn Sprague-Dawley rats (P0-P7), regardless of gender, following the National Institutes of Health’s Guide for the Care and Use of Laboratory Animals. The dissociated cells were maintained in a Neurobasal medium (Gibco, 21103049, USA) with 2% B27 supplement (Gibco, A3582801, USA), 1% L-Glutamine (Gibco, 25030081, USA), and 1% Penicillin-Streptomycin-Amphotericin B Solution (Beyotime, C0224, China) at 37°C, 5% CO_2_. In culture,1/2 of the medium was changed every 2-3 days for up to 2 weeks.

**Immunofluorescence**

Primary cells were seeded in confocal dishes. After 48 hours of transfection, the primary cells were fixed with 4% formaldehyde for 15 min, permeabilized with 0.1% Triton X-100 for 20 min, and blocked with 5% BSA for 30 min. The cells were incubated with primary antibodies, including HTR1B (Abcam, ab85937, UK), Arc (Proteintech, 16290-1-AP, China), TPH2 (Abcam, ab288067, UK) at 4℃ overnight and then incubated with the secondary antibodies FITC (Abclonal, AS011, China) or Cy3 (Abclonal, AS007, China) at room temperature for 1 h before staining with DAPI. The fluorescence signal was captured by a Leica microscope using × 40 objective lenses.

**Morphology and spine density**

To study the morphology of 5-HT neurons and spine density, rat DRN was plated on the Poly-D-lysine (PDL) hydrobromide (50 µg/mL) pre‐coated confocal dishes (35 mm). We transfected ERVWE1, siHTR1B, or siALKBH5 into DRN at 14 days in *vitro* (DIV14).

We examined the complexity of 5-HT neurons by Sholl analysis on DIV17. Cells were fixed with 4% formaldehyde, permeabilized with 0.1% Triton X-100, blocked with 5% BSA, and incubated with MAP2 (Abclonal, A22206, China) at 4℃ overnight. After washing, Cy3 (Abclonal, AS007, China) was added to the cells, and then the images were observed using a Leica microscope with × 40 objective lenses.

In order to investigate the spine density, cells were fixed with 4% formaldehyde three days post-transfection. Immunofluorescence with FITC Phalloidin (Abclonal, RM02836, China) was observed using a Leica microscope using × 60 oil objective lens. We used Image J software to analyze the spine density of 5-HT neurons. The spine types were determined by the specific criteria of spines (mushroom: spine head width > spine neck width*2, thin: spine head width ≥ spine neck width, stubby: spine length < spine head width*2.5, filopodia: spine length > spine head width*2.5).

**M6A dot blot and quantification assay**

Total RNA was extracted from the SH-SY5Y cell line using TRIzol (Invitrogen, 15596026, USA) and spotted onto two nitrocellulose membranes (RPN303B, Amersham, USA). After UV cross-linked to the membrane, one membrane was probed with an anti-m6A antibody (abclonal, A19841, China), and the other membrane was stained with Methylene blue (MB) as a control. The EpiQuik m6A RNA Methylation Quantification Kit (Colorimetric) (Epigentek, P9005, USA) was used to quantify the global m6A levels according to the manufacturer’s protocol.

**Me-RIP-qPCR**

600 μg of total RNA (dissolve 300 μg RNA in 300 μL IP buffer: 150 mmol/L NaCl, 0.1% NP-40, 10 mmol/L Tris-HCl, pH 7.4) was enriched with an m6A-antibody (abclonal, A19841, China) and IgG (abclonal, AC005, China, as a control) and subsequently immunoprecipitated with Protein A/G Magnetic Beads supplemented with RNase inhibitors at 4°C overnight. After immunoprecipitation, we used TRIzol (Invitrogen, 15596026, USA) to isolate RNA and detected mRNA levels by RT-qPCR. The specific primers for HTR1B were listed in Table S7.

**RNA immunoprecipitation (RIP)**

SH-SY5Y cells were treated with 1% formaldehyde for 10 min and 2.5 mmol/L glycine for 5 min. Afterward, we added RIP buffer (150 mmol/L KCl, 25 mmol/L Tris–HCl pH 7.4, 5 mmol/L EDTA, 0.5 mmol/L DTT, 0.5% NP-40, 100 U/mL RNase inhibitors, 100 µmol/L PMSF, 100 µg/mL protease Inhibitor) to lyse cells and prepared them followed by sonication. Protein A/G magnetic beads conjugated with control rabbit IgG (abclonal, AC005, China) and ALKBH5 (Proteintech, 16837-1-AP, China) antibodies were incubated at 4°C overnight with cell lysates. RNA–protein complexes were treated with proteinase K buffer. Finally, RNA was extracted using the TRIzol reagent (Invitrogen, 15596026, USA) and analyzed by RT-qPCR.

**RNA stability**

Cells were transfected after 24 h and then treated with Actinomycin D 5 µg/mL at 6, 3, and 0 h. Total RNA was extracted using TRIzol (Invitrogen, 15596026, USA), and the relative mRNA expression levels were measured using RT-qPCR with GAPDH normalization. We used GraphPad Prism 8.0 to calculate the mRNA decay rate by nonlinear regression curve fitting (one-phase decay model).

**Luciferase Reporter Assay**

For the promoter assay, cells were seeded in a 24-well plate and tri-transfected with ERVWE1 expressed plasmids (pcDNA3.1/pcDNA3.1-ERVWE1), ALKBH5 promoter constructs (pGL3-basic/ pGL3-ALKBH5), and a Renilla plasmid pRL-TK. After 24 hours of transfection, cells were lysed, and luciferase activity was measured using the Promega Dual-Luciferase Reporter Assay (Promega, E1960, USA) according to the manufacturer’s instructions.

To investigate the potential m6A modified sites, cells were seeded in a 24-well plate and co-transfected with pcDNA3.1/pcDNA3.1-ERVWE1/PXJ40/ PXJ40-ALKBH5 and pmirGLO plasmids fused with wild-type and mutated motifs of HTR1B. After 36 hours of transfection, the relative luciferase activity (Firefly luciferase/Renilla luciferase) was measured by a dual-luciferase reporter assay system (Promega, E1960, USA) according to the manufacturer’s protocol. Moreover, the Firefly luciferase/Renilla luciferase ratio was assessed at 48 h transfection after siNC/siALKBH5 treatment.

**References:**

1. Li X, Wu X, Li W, Yan Q, Zhou P, Xia Y et al (2023) HERV-W ENV Induces Innate Immune Activation and Neuronal Apoptosis via linc01930/cGAS Axis in Recent-Onset Schizophrenia. Int J Mol Sci 24:3000.

**Supplementary Tables**

**Table S1.** The concentration of SERT in the plasma of healthy controls and schizophrenia patients

| Healthy controls (*n* = 37, pg/mL) | | Schizophrenia patients (*n* = 44, pg/mL) | |
| --- | --- | --- | --- |
| Mean | 1415.5676 | Mean | 693.1682 |
| Median | 1403 | Median | 402.2 |
| Std. Deviation | 492.27592 | Std. Deviation | 481.34571 |
| Skewness | -0.034 | Skewness | 0.558 |
| Sta. Error of Skewness | 0.388 | Sta. Error of Skewness | 0.357 |
| Range | 2250 | Range | 1598.8 |
| Minimum | 243 | Minimum | 159.2 |
| Maximum | 2493 | Maximum | 1758 |

**Table S2.** The concentration of TPH2 in the plasma of healthy controls and schizophrenia patients

| Healthy controls (*n* = 37, pg/mL) | | Schizophrenia patients (*n* = 44, pg/mL) | |
| --- | --- | --- | --- |
| Mean | 32254.9856 | Mean | 14899.7683 |
| Median | 29696.6019 | Median | 7995.1456 |
| Std. Deviation | 14311.85462 | Std. Deviation | 13984.58435 |
| Skewness | 1.612 | Skewness | 1.107 |
| Sta. Error of Skewness | 0.388 | Sta. Error of Skewness | 0.357 |
| Range | 69417.48 | Range | 54638.35 |
| Minimum | 7730.58 | Minimum | 1393.2 |
| Maximum | 77148.06 | Maximum | 56031.55 |

**Table S3.** The concentration of 5-HT in the plasma of healthy controls and schizophrenia patients

| Healthy controls (*n* = 37, pg/mL) | | Schizophrenia patients (*n* = 44, pg/mL) | |
| --- | --- | --- | --- |
| Mean | 2875.6757 | Mean | 1201.9697 |
| Median | 2550 | Median | 547.5 |
| Std. Deviation | 1182.71433 | Std. Deviation | 1178.2439 |
| Skewness | 1.762 | Skewness | 1.628 |
| Sta. Error of Skewness | 0.388 | Sta. Error of Skewness | 0.357 |
| Range | 6758.33 | Range | 5510 |
| Minimum | 833.33 | Minimum | 140 |
| Maximum | 7591.67 | Maximum | 5650 |

**Table S4.** The concentration of ERVWE1 in the plasma of healthy controls and schizophrenia patients

| Healthy controls (*n* = 37, pg/mL) | | Schizophrenia patients (*n* = 44, pg/mL) | |
| --- | --- | --- | --- |
| Mean | 1692.7568 | Mean | 2989.2727 |
| Median | 1296 | Median | 2712 |
| Std. Deviation | 1052.89013 | Std. Deviation | 1321.80786 |
| Skewness | 1.14 | Skewness | 0.908 |
| Sta. Error of Skewness | 0.388 | Sta. Error of Skewness | 0.357 |
| Range | 3840 | Range | 5024 |
| Minimum | 592 | Minimum | 1048 |
| Maximum | 4432 | Maximum | 6072 |

**Table S5.** Comparison of the whole peripheral blood samples demographic data between the healthy controls and recent-onset schizophrenia patients

|  | Schizophrenia patients (*n* = 15) | | Healthy controls  (*n* = 14) | | Analysis |
| --- | --- | --- | --- | --- | --- |
|  | Median | Range | Median | Range | *p* |
| Age (years)^a^ | 45 | 29-66 | 42 | 29-64 | 0.914 |
| Education (years) ^a^ | 12 | 3-16 | 9 | 5-16 | 0.252 |
| BMI (body mass index) ^a^ | 20.5 | 16.9-26.4 | 19.1 | 15.3-26.7 | 0.847 |
|  | N | % | N | % | *p* |
| Gender ^b^ |  |  |  |  |  |
| Male | 7 | 47 | 6 | 43 | 0.837 |
| Female | 8 | 53 | 8 | 57 |  |
| Smoking status ^b^ |  |  |  |  |  |
| Yes | 5 | 33 | 6 | 43 | 0.597 |
| No | 10 | 67 | 8 | 57 |  |

Notes: a: *p* values were calculated by the Mann-Whitney U test.

b: *p* values were calculated by the chi-square test.

**Table S6.** Comparison of the plasma samples demographic data between the healthy controls and recent-onset schizophrenia patients

|  | Schizophrenia patients (*n* = 44) | | Healthy controls  (*n* = 37) | | Analysis |
| --- | --- | --- | --- | --- | --- |
|  | Median | Range | Median | Range | *p* |
| Age (years)^a^ | 42.5 | 22-66 | 41 | 23-65 | 0.936 |
| Education (years) ^a^ | 12 | 3-16 | 12 | 3-16 | 0.671 |
| BMI (body mass index) ^a^ | 22.0 | 16.9-26.4 | 22.6 | 15.6-27.5 | 0.872 |
|  | N | % | N | % | *p* |
| Gender ^b^ |  |  |  |  |  |
| Male | 21 | 48 | 17 | 46 | 0.873 |
| Female | 23 | 52 | 20 | 54 |  |
| Smoking status ^b^ |  |  |  |  |  |
| Yes | 19 | 43 | 16 | 43 | 0.996 |
| No | 25 | 57 | 21 | 57 |  |

Notes: a: *p* values were calculated by the Mann-Whitney U test.

b: *p* values were calculated by the chi-square test.

**Table S7.** Primer sequences used in real-time quantitative PCR (RT-qPCR)

| Name | Primer sequence (5’ – 3’) |
| --- | --- |
| ERVWE1-F | CCATGCCGCTGTATGACCAG |
| ERVWE1-R | GGGTTCCCTTAGAAAGACTCCT |
| GAPDH-F | ATGACATCAAGAAGGTGGTG |
| GAPDH-R | CATACCAGGAAATGAGCTTG |
| HTR1A-F | ACCATTAGCAAGGATCATGGC |
| HTR1A-R | ATATGCGCCCATAGAGAACCA |
| HTR1B-F | CAGCGCCAAGGACTACATTT |
| HTR1B-R | ACAAAGGCATTGGAGAGCGT |
| HTR2A-F | CACACGGGCCAAATTAGCTT |
| HTR2A-R | CACCTTGCATGCCTTTTGCT |
| HTR6-F | GCAACACGTCCAACTTCTTCC |
| HTR6-R | TGCAGCACATCACGTCGAA |
| HTR7-F | CGAAGATGATTCTCTCCGTCTG |
| HTR7-R | GCGGTAGAGTAAATCGTATAGCC |
| Arc-F | TGGACACGCAGATCTTCGAG |
| Arc-R | TCACGGAGCCCTGCTTGAAC |
| METTL3-F | TTGTCTCCAACCTTCCGTAGT |
| METTL3-R | CCAGATCAGAGAGGTGGTGTAG |
| METTL14-F | GTAGCACAGACGGGGACTTC |
| METTL14-R | TTGGTCCAACTGTGAGCCAG |
| FTO-F | TCTCATCTCGAAGGCAGGGA |
| FTO-R | GGTGGGTGGCATTGAGATCA |
| ALKBH5-F | CGGCGAAGGCTACACTTACG |
| ALKBH5-R | CCACCAGCTTTTGGATCACCA |
| YTHDF1-F | GGGGACAAGTGGGTCTCAAG |
| YTHDF1-R | GGGGACAAGTGGGTCTCAAG |
| YTHDF2-F | GTTGGTAGCGGGTCCATTACT |
| YTHDF2-R | GGTCTTCAGTTTAGGTTGCTGT |
| YTHDF3-F | ATCAGAGTAACAGCTATCCAC |
| YTHDF3-R | CCCAGGTTGACTAAATACAC |
| YTHDC1-F | AACTGGTTTCTAAGCCACTGAGC |
| YTHDC1-R | GGAGGCACTACTTGATAGACGA |
| YTHDC2-F | ATCGCTGTGGCTGAAAGAGT |
| YTHDC2-R | TCCTGCCATCAATGTACGAA |

**Table S8.** Primer sequences used in plasmid constructs and siRNA

| Name | Primer sequence (5’ – 3’) |
| --- | --- |
| PXJ40-ALKBH5-F | CCCAAGCTTCGGAGGACCCTAGAGCAGCGTC |
| PXJ40-ALKBH5-R | CGGGGTACCTCAGTGCCGCCGCATCTTCACC |
| pcDNA3.1(-)-HTR1B-F | GGAATTCATGGAGGAACCGGGTGCTCAGTGC |
| pcDNA3.1(-)-HTR1B-R | CCCAAGCTTACTTGTGCACTTAAAACGTATCAG |
| HTR1B-Mut198-F | GCGCCAAGGATTACATTTACCAGGACTCCAT |
| HTR1B-Mut198-R | TAAATGTAATCCTTGGCGCTGCAGTTTTGGG |
| HTR1B-Mut572-F | GCTAAAAGGACGCCCAAGAGGGCGGCG |
| HTR1B-Mut572-R | CTTGGGCGTCCTTTTAGCTGAGTACTCCAC |
| HTR1B-Mut1212-F | CAATGAGGATTTTAAACAAGCATTCCAT |
| HTR1B-Mut1212-R | GTTTAAAATCCTCATTGGACATGGTATAGA |
| pIMRHTR1B-F | CTAGCTAGCATGGAGGAACCGGGTGCTC |
| pIMRHTR1B-R | GCTCTAGAACTTGTGCACTTAAAACGTATC |
| pGL3-ALKBH5-F | CGGGGTACCCGCCCGTTCGTTTCCTGGAG |
| pGL3-ALKBH5-R | CCCAAGCTTCTCGGGGTCCGAGTCCTCCT |
| siHTR1B | GTATGTGAACCAAGTCAAA |
| siHTR1B-NC | UUCUCCGAACGUGUCACGUTT |
| siALKBH5 | CCTCAGGAAGACAAGATTAGA |
| siALKBH5-NC | UUCUCCGAACGUGUCACGUTT |

**Table S9.** Antibodies used in western blot

| Antibodies | Manufacturers | Cat No. |
| --- | --- | --- |
| ERVWE1 Rabbit pAb | Abclonal | A16522 |
| Anti-HTR1B | Abcam | ab85937 |
| ERK1/2 Polyclonal antibody | Proteintech | 16443-1-AP |
| Phospho-ERK1/2 (Thr202/Tyr204) | Proteintech | 28733-1-AP |
| ELK1 Rabbit mAb | Abclonal | A19046 |
| Phospho-ELK1-S383 Rabbit pAb | Abclonal | AP0033 |
| Arc Polyclonal antibody | Proteintech | 16290-1-AP |
| METTL3 Rabbit mAb | Abclonal | A19079 |
| METTL14 Rabbit pAb | Abclonal | A8530 |
| FTO Rabbit mAb | Abclonal | A3861 |
| YTHDC2 Rabbit pAb | Abclonal | A15004 |
| ALKBH5 Polyclonal antibody | Proteintech | 16837-1-AP |
| Anti-TPH2 | Abcam | ab288067 |
| GAPDH Mouse mAb | Abclonal | AC002 |
| N6-methyladenosine(m6A) Rabbit mAb | Abclonal | A19841 |
| Mouse anti Myc-Tag mAb | Abclonal | AE010 |
| HRP Goat Anti-Mouse IgG | Abclonal | AS003 |
| HRP Goat Anti-Rabbit IgG | Abclonal | AS014 |

**Supplementary Figures**


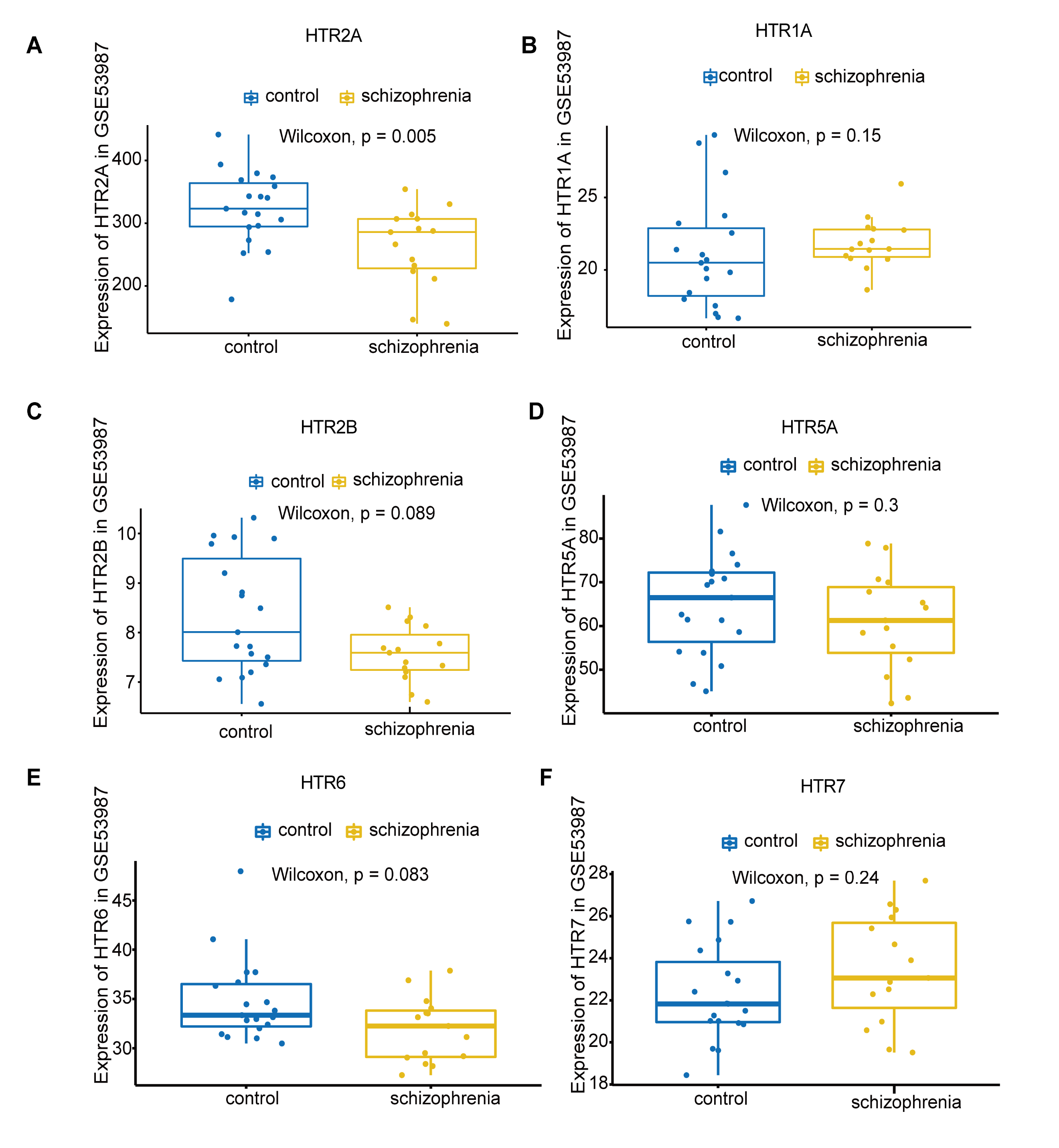


**Fig. S1** 5-HT receptors abnormality in the prefrontal cortex (BA46) of schizophrenia by GSE53987. **A**-**F** Boxplot of 5-HT receptors expression in schizophrenia (*n* = 15) vs healthy controls (*n* = 19), **A** Boxplot of HTR2A expression, **B** Boxplot of HTR1A expression, **C** Boxplot of HTR2B expression, **D** Boxplot of HTR5A expression, **E** Boxplot of HTR6 expression, **F** Boxplot of HTR7 expression. *P*-valve was analyzed by wilcoxon (Mann-Withney). Data shown are the mean ± SD.


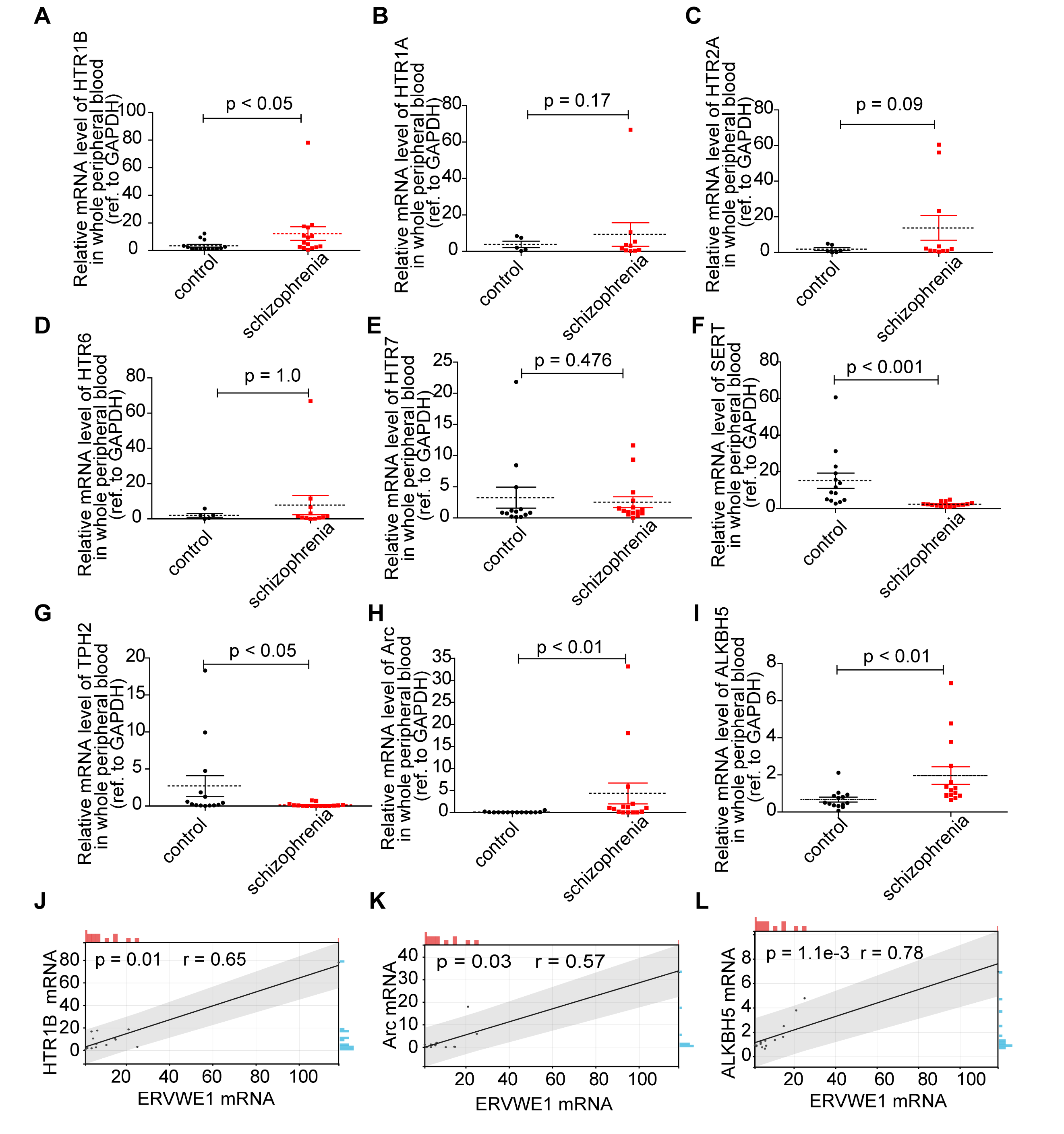


**Fig. S2** mRNA expression level of 5-HTergic systems, Arc, and ALKBH5 in the whole peripheral blood of schizophrenia patients and healthy controls. **A**-**I** Respectively represent the mRNA expression levels of HTR1B, HTR1A, HTR2A, HTR6, HTR7, SERT, TPH2, Arc, and ALKBH5 in the whole peripheral blood of schizophrenia patients (*n* = 15) and healthy controls (*n* = 14) by RT-qPCR (*p* - value by median and nonparametric analysis). **J**-**L** Correlation of ERVWE1 mRNA level with HTR1B (*p* = 0.01, r = 0.65), Arc (*p* = 0.03, r = 0.57), and ALKBH5 (*p* < 0.01, r = 0.78) mRNA levels in schizophrenia by Spearman. Dots depict schizophrenia patients, but a few are overlapping and cannot be separated on the graph. Data shown are the mean ± SD.


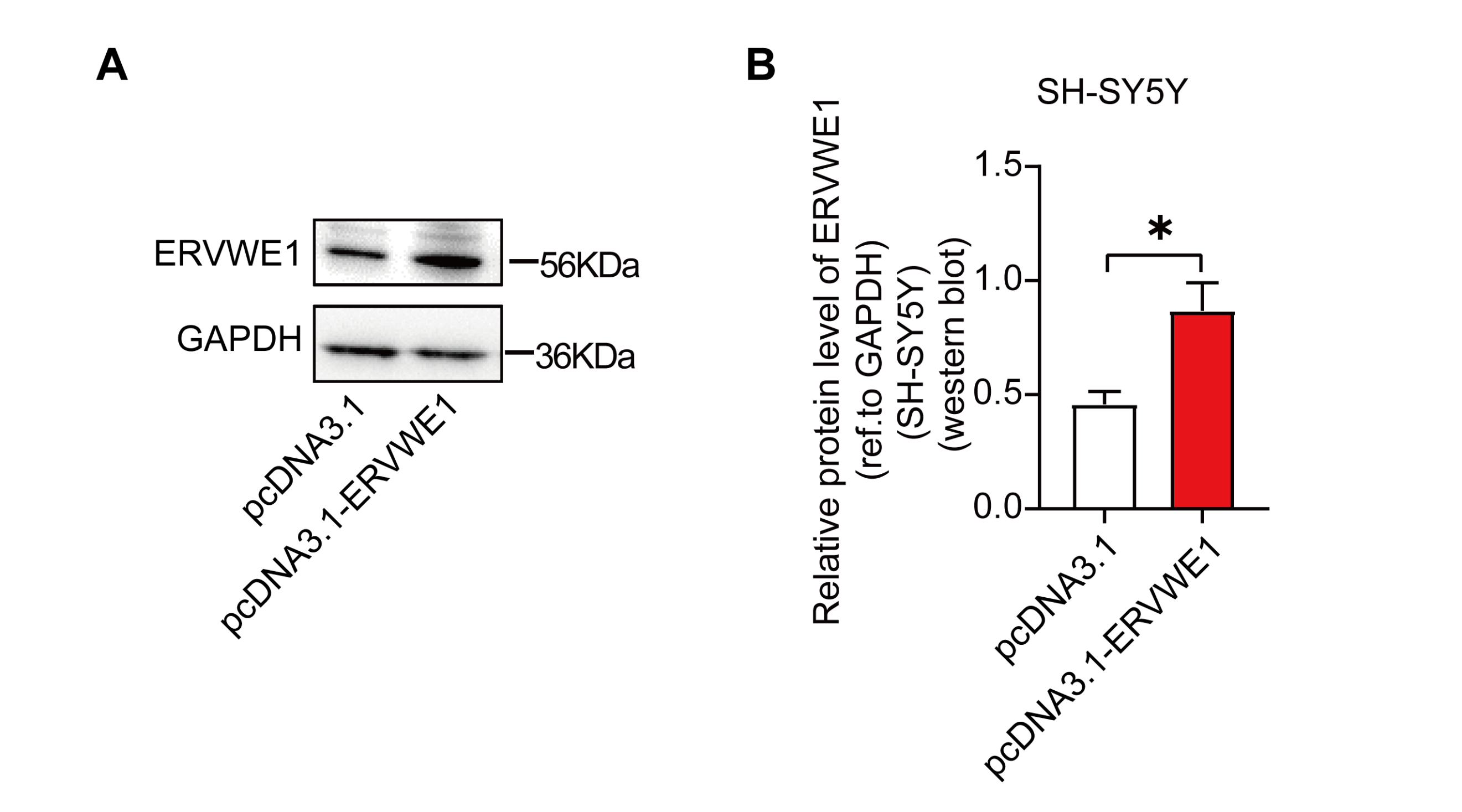


**Fig. S3** ERVWE1 overexpressed in the SH-SY5Y cells. **A** The representative western blot results of ERVWE1. **B** Protein expression level of ERVWE1. Data shown are the mean ± SD and represent three independent experiments. Statistical analysis: Student’s *t*-test (**p* < 0.05).

**
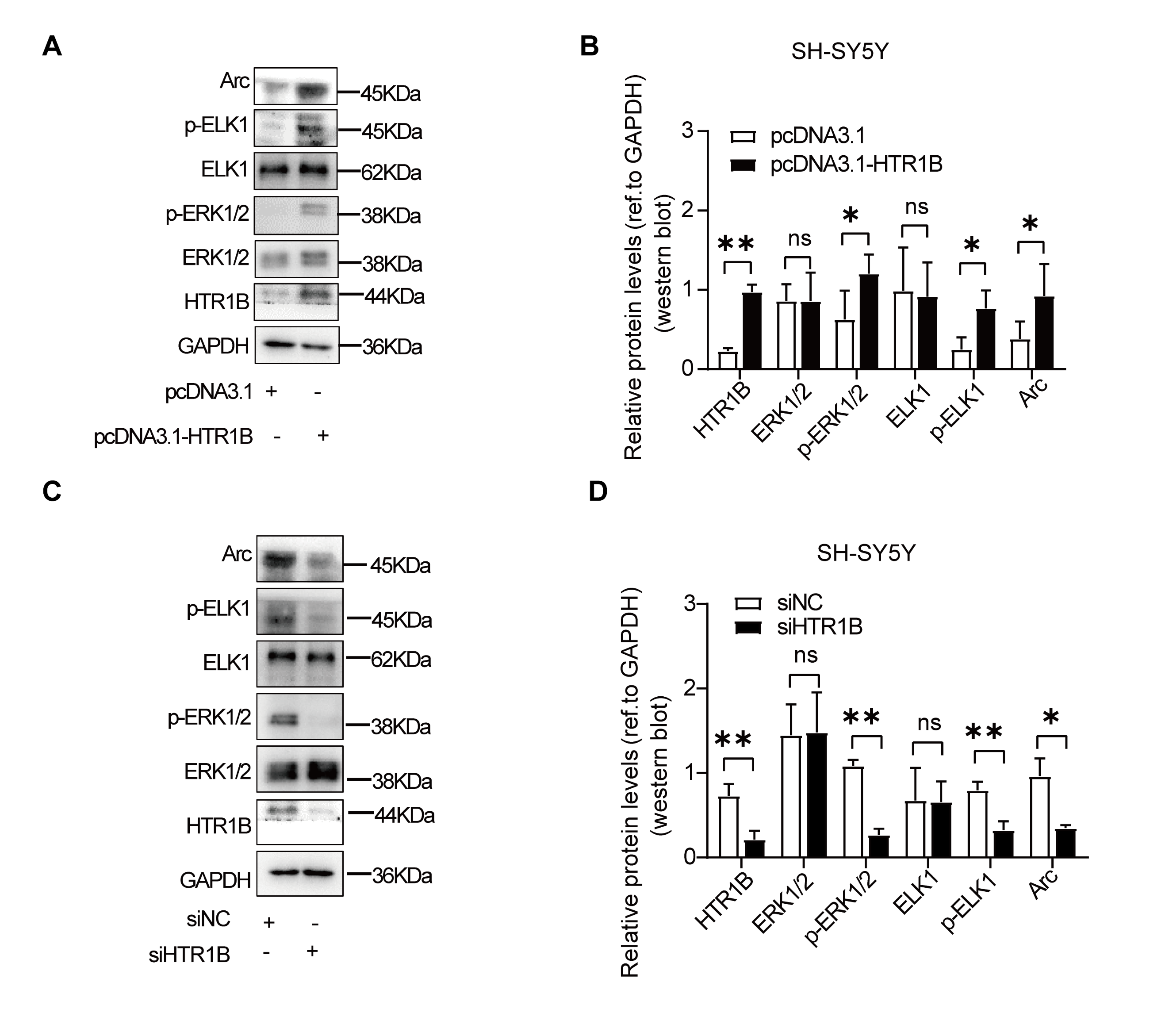
**

**Fig. S4** HTR1B activated ERK-ELK1-Arc signal pathway in the SH-SY5Y cells. **A**-**D** Representative western blot results for ERK1/2, p-ERK1/2, ELK1, p-ELK1, and Arc proteins in the SH-SY5Y cells after transfection with HTR1B expressed vectors and siRNA oligo respectively. Data shown are the mean ± SD and represent three independent experiments. Statistical analysis: Student’s *t*-test (^ns^ *p* > 0.05, **p* < 0.05, ***p* < 0.01).


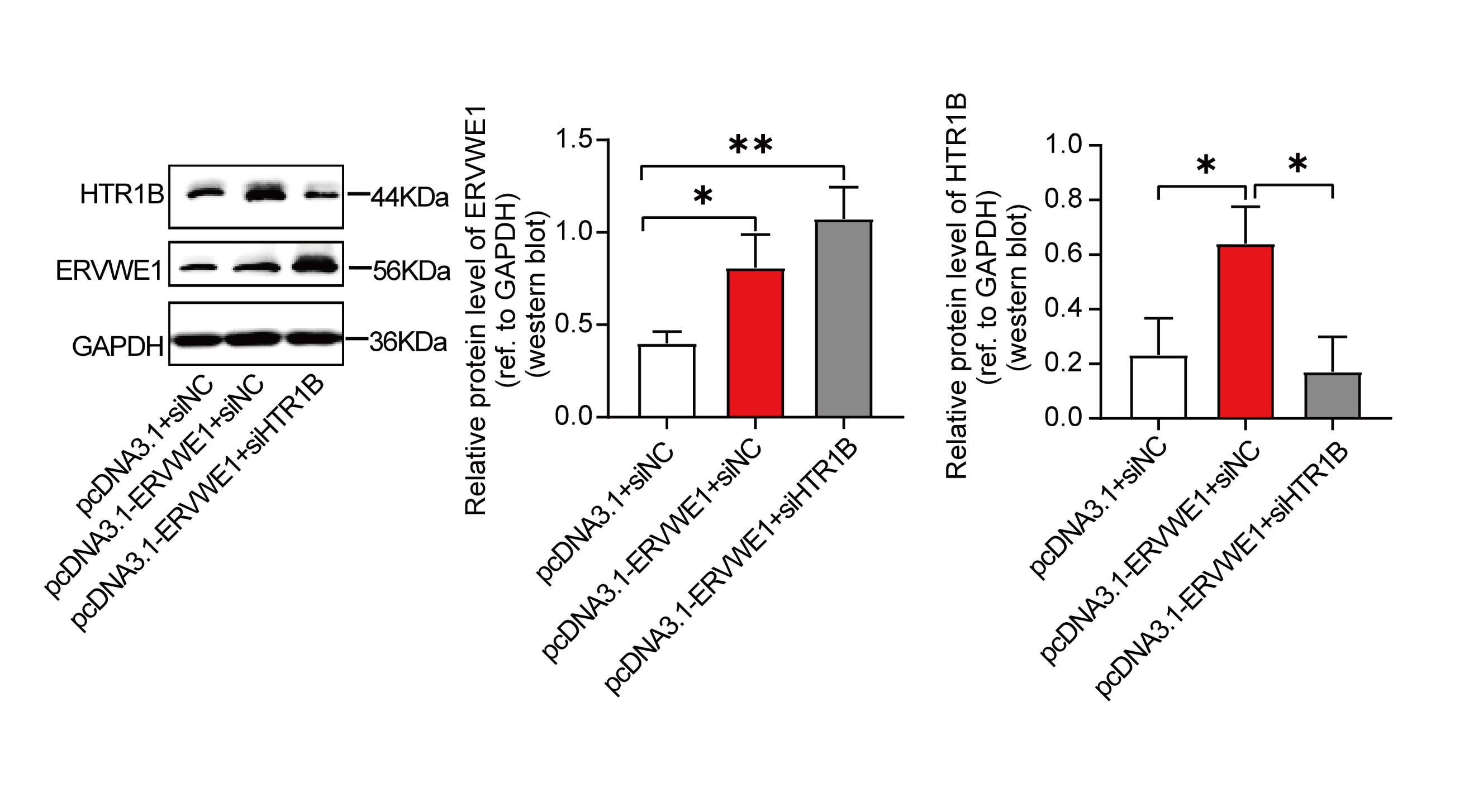


**Fig. S5** ERVWE1 activated HTR1B signal pathway in SH-SY5Y cells. The protein expression levels of ERVWE1 and HTR1B were detected in cells co-transfected with ERVWE1 and siHTR1B. Data shown are the mean ± SD and represent three independent experiments. Statistical analysis: one-way ANOVA (**p* < 0.05, ***p* < 0.01).


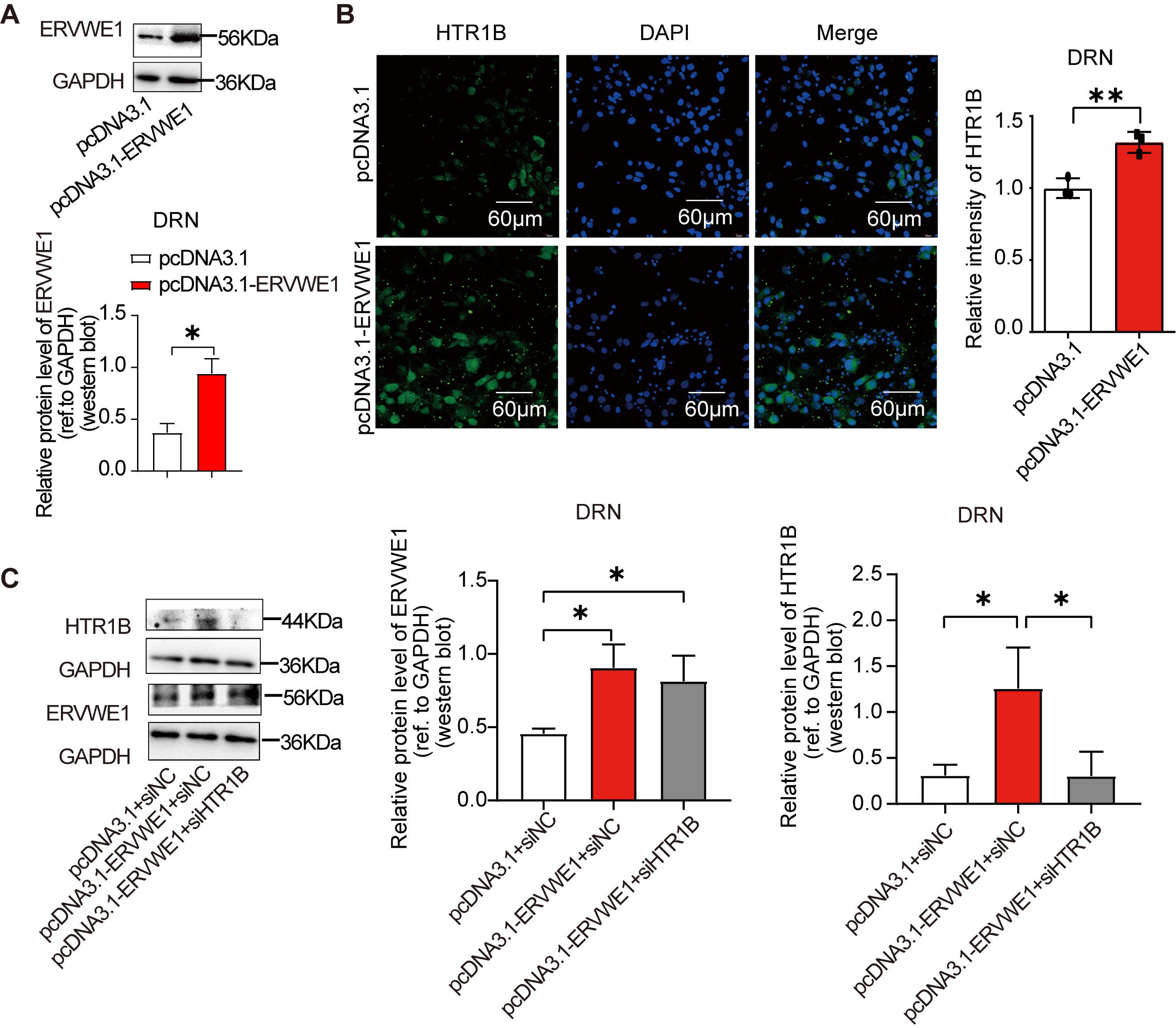


**Fig. S6** ERVWE1 activated HTR1B signal pathway in DRN neurons. **A** Transfection efficiency of ERVWE1 in DRN neurons by western blot. **B** Immunofluorescence staining with HTR1B in DRN neurons. **C** The western blot results of ERVWE1 and HTR1B were detected by applying siHTR1B and ERVWE1. Data shown are the mean ± SD and represent three independent experiments. Statistical analysis: Student’s *t*-test and one-way ANOVA (**p* < 0.05, ***p* < 0.01).

**
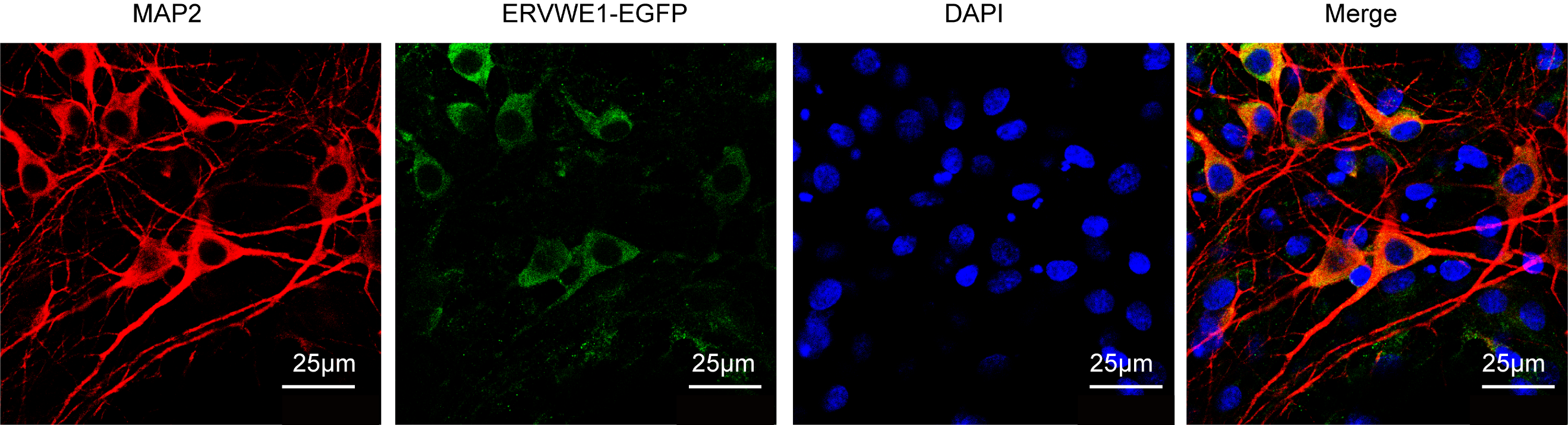
**

**Fig. S7** The transfection efficiency of ERVWE1 in the DRN neurons. Scale bars = 25 μm.


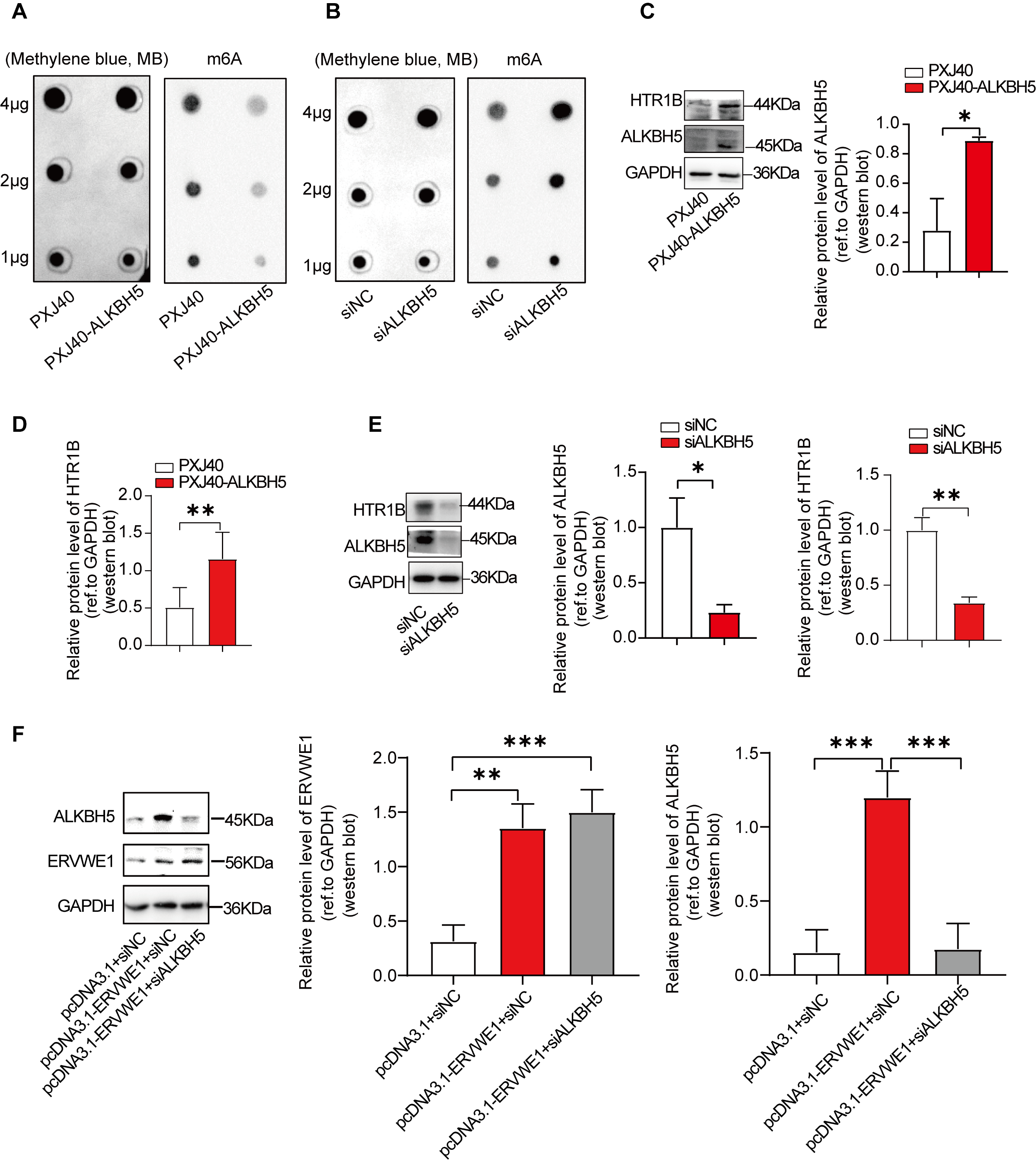


**Fig. S8** ALKBH5 regulated m6A modification in SHSY5Y. **A**, **B** RNA dot blot analysis of m6A levels. **A** ALKBH5 expressed cells vs control cells, **B** siALKBH5 cells and siNC cells. Methylene blue staining served as a loading control. **C**-**E** Western blot results of ALKBH5 and HTR1B. **C, D** ALKBH5 overexpression, **E** ALKBH5 knockdown. **F** The western blot results of ERVWE1 and ALKBH5 were detected when ERVWE1 co-transfected with siALKBH5 or siNC control. Data shown are the mean ± SD and represent three independent experiments. Statistical analysis: Student’s *t*-test and one-way ANOVA (**p* < 0.05, ***p* < 0.01, ****p* < 0.001).


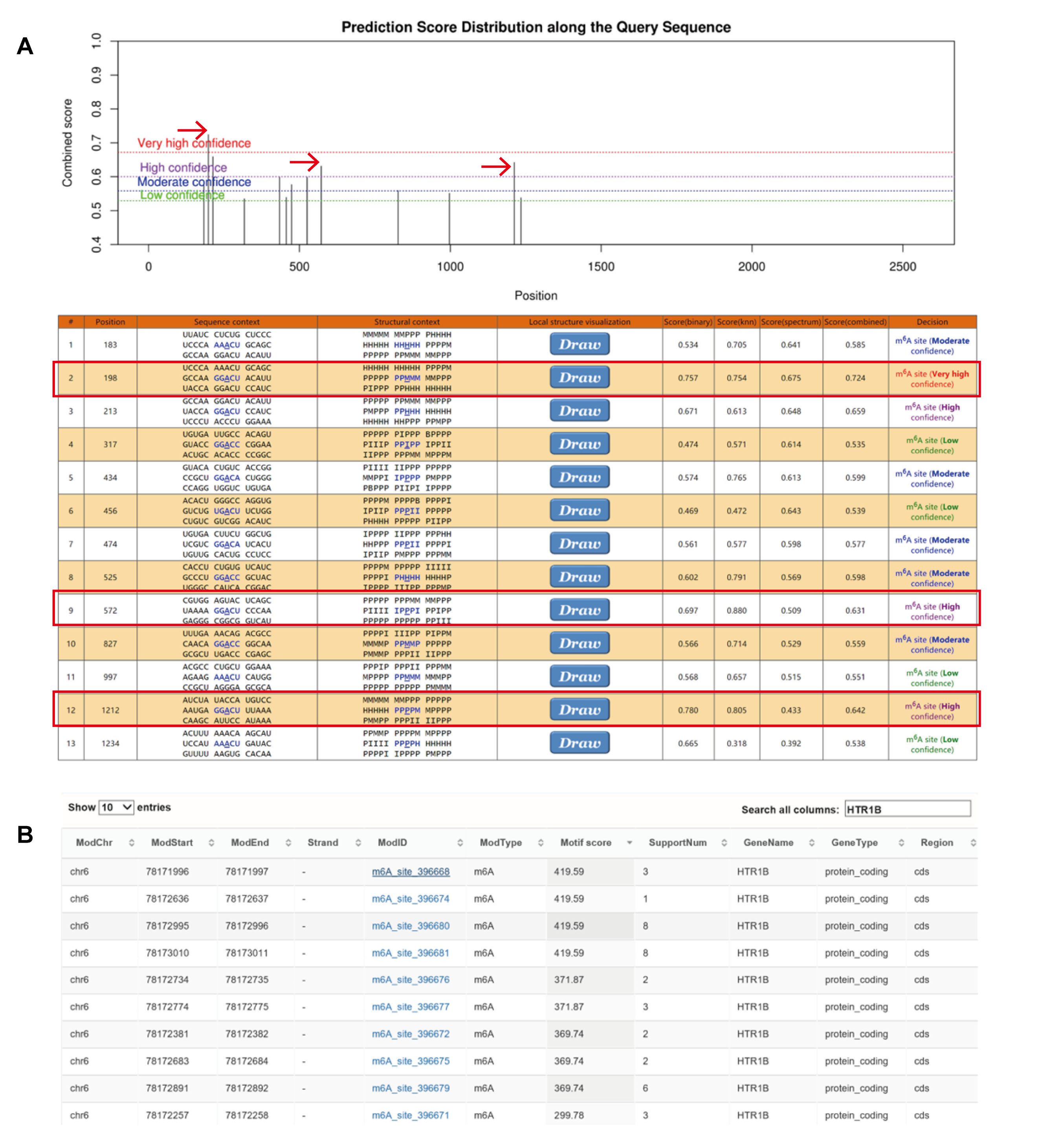


**Fig. S9** m6A modification sites predictor of HTR1B mRNA. **A** SRAMP. High and very high confidences were considered as a candidate site. **B** RMBase v2.0. Score above 400 was considered as a candidate site.


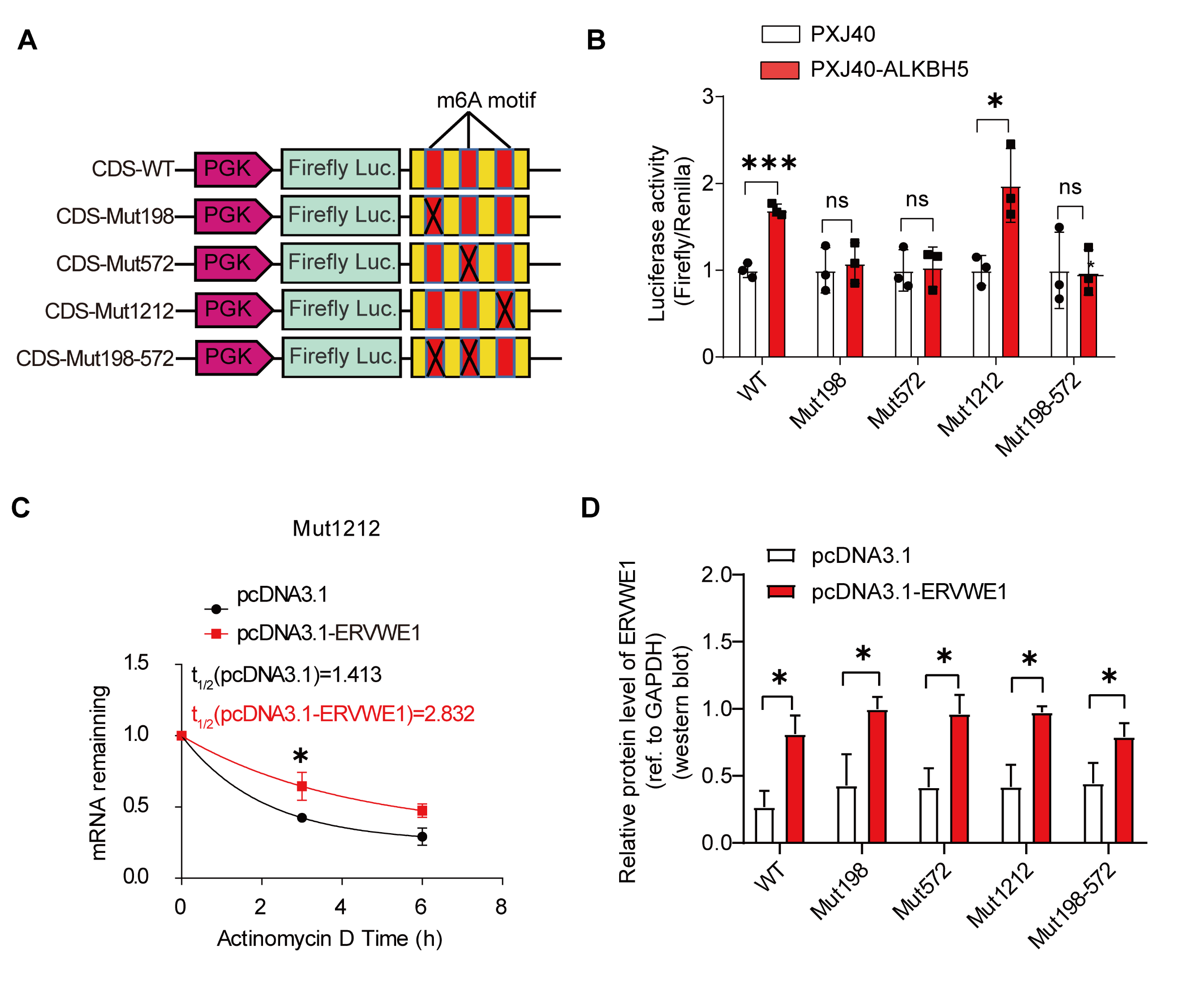


**Fig. S10** ERVWE1 reduced the m6A level of HTR1B by 198 and 574 sites. **A** Schematic illustration of HTR1B with either wild-type or mutant m6A sites luciferase activity plasmids constructs. **B** Relative luciferase activity of HTR1B-CDS wild-type and mutant m6A sites after co-transfection with ALKBH5 expressing plasmid or control plasmid in SH-SY5Y cells. **C** Decay of HTR1B mRNA with ERVWE1 overexpression versus control in the HTR1B-CDS-Mut1212 groups in SH-SY5Y cells. **D** The relative protein levels of ERVWE1 in SH-SY5Y cells. Data shown are the mean ± SD and represent three independent experiments. Statistical analysis: Student’s *t*-test (^ns^ *p* > 0.05, **p* < 0.05, ****p* < 0.001).


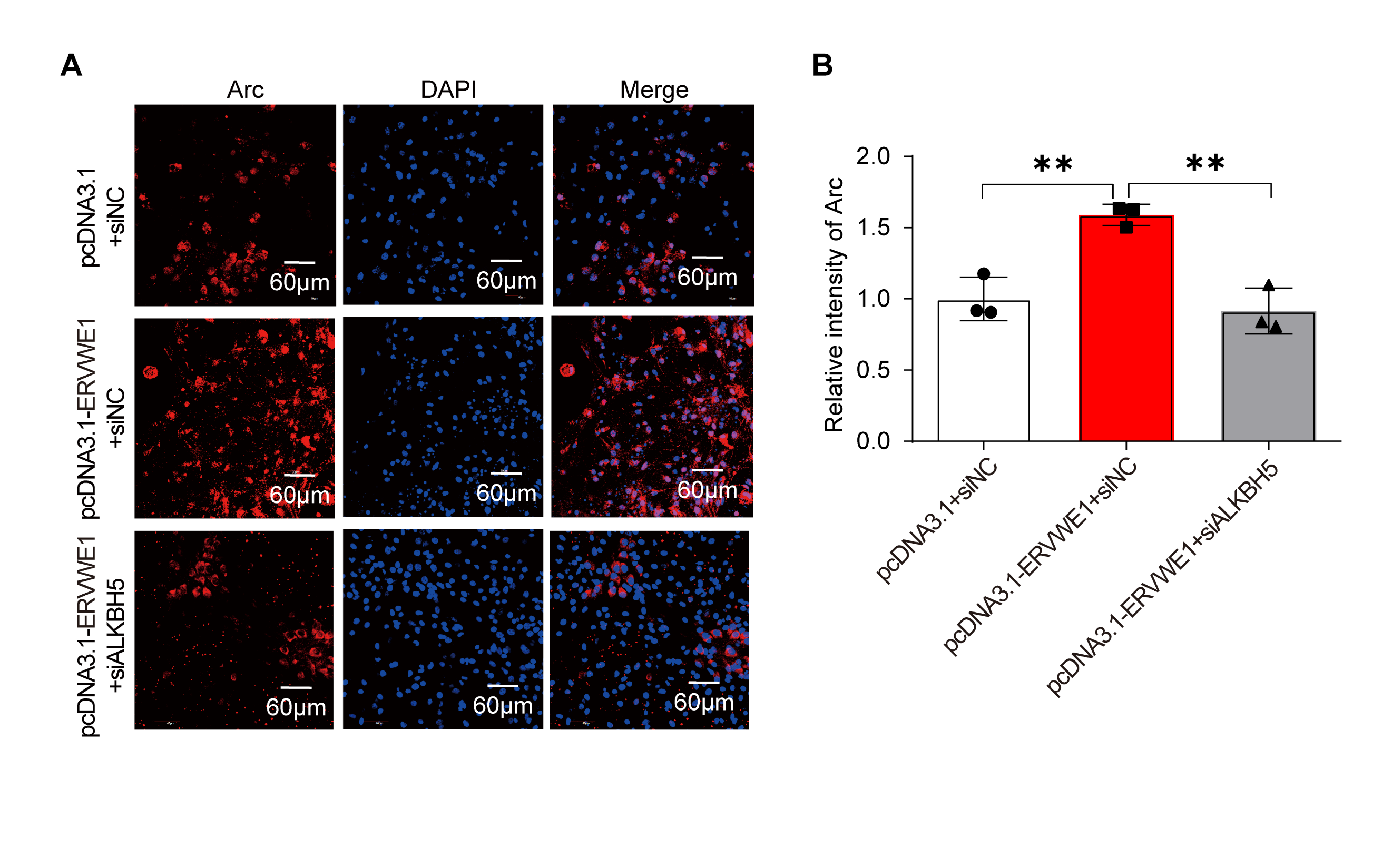


**Fig. S11** ALKBH5 participated in ERVWE1-induced Arc upregulation in DRN neurons. **A** Immunofluorescence staining with Arc in DRN neurons. **B** The relative intensity of Arc. Data shown are the mean ± SD and represent three independent experiments. Statistical analysis: one-way ANOVA (***p* < 0.01).
